# Contrasting diversity of vaginal lactobacilli among the females of Northeast India

**DOI:** 10.1186/s12866-019-1568-6

**Published:** 2019-08-27

**Authors:** Sumi Das Purkayastha, Mrinal Kanti Bhattacharya, Himanshu Kishore Prasad, Hrishikesh Upadhyaya, Suparna Das Lala, Kunal Pal, Meenakshi Das, Gauri Dutt Sharma, Maloyjo Joyraj Bhattacharjee

**Affiliations:** 1 Karimganj College, Karimganj, Assam India; 2 Department of Life Science and Bioinformatics, Assam University, Silchar, Assam India; 3 Department of Botany, Cotton University, Guwahati, Assam India; 4 Department of Gynaecology, Hospital of Red Cross Society, Karimganj, Assam India; 5 Department of Biotechnology and Medical Engineering, NIT Rourkela, Rourkela, Orissa India; 6 Bilaspur University, Bilaspur, Chhattisgarh India

**Keywords:** Vaginal microbiota, *Lactobacillus*, Northeast India, Non-pregnant and pregnant women

## Abstract

**Background:**

Lactobacilli are gatekeepers of vaginal ecosystem impeding growth of pathogenic microbes and their diversity varies across populations worldwide. The present study investigated diversity of human vaginal microbiota among females of Northeast India, who are distinct in dietary habits, lifestyle, and genomic composition from rest of India.

**Results:**

Altogether, 154 bacterial isolates were obtained from vaginal swab samples of 40 pregnant and 29 non-pregnant females. The samples were sequenced for 16 s rRNA gene and analysed for identification using a dual approach of homology search and maximum likelihood based clustering. Molecular identification based on 16S rRNA gene sequence confirmed the isolates belonging to 31 species. Lactobacilli constituted 37.7% of the bacterial isolates with 10 species and other Lactic Acid Bacteria (39.61%) represented another 10 species, some of which are opportunistic pathogens. The remaining of the communities are mostly dominated by species of *Staphylococcus* (14.28%) and rarely by *Propionibacterium avidum* (3.90%)*, Bacillus subtilis, Escherchia coli, Janthinobacterium lividum,* and *Kocuria kristinae* (each 0.64%). Interestingly *Lactobacillus mucosae* and *Enterococcus faecalis,* which are globally uncommon vaginal microbes is found dominant among women of Northeast India. This tentatively reflects adaptability of particular *Lactobacillus* species, in distinct population, to better compete for receptors and nutrients in vaginal epithelium than other species. Further, intrageneric 16S rRNA gene exchange was observed among *Enterococcus, Staphylococcus*, and two species of *Lactobacillus*, and deep intraspecies divergence among *L. mucosae,* which pinpointed possibility of emergence of new strains with evolved functionality. Lactobacilli percentage decreased from young pregnant to aged non-pregnant women with maximum colonization in trimester II.

**Conclusion:**

The study highlighted importance of assessment of vaginal microbiota, *Lactobacillus* in particular, across different population to gain more insight on female health.

**Electronic supplementary material:**

The online version of this article (10.1186/s12866-019-1568-6) contains supplementary material, which is available to authorized users.

## Background

Lactobacilli are the predominant microbes in the vaginal microenvironment of healthy females [1]. A key feature that favours colonization of *Lactobacillus* is low vaginal pH due to metabolism of glycogen to lactic acid; glycogen deposits in vaginal epithelial cells in response to rise in circulating estrogen [2, 3]. The predominance of *Lactobacillus* promotes vaginal health by inhibiting colonization of pathogenic microbes at acidic pH and also by producing antimicrobial bacteriocins [4]. However, the vaginal pH and *Lactobacillus* levels vary over the life course of women [5]. During bacterial vaginosis (BV), *Lactobacillus* predominance is lost and replaced by pathogenic microbes, which adversely affects women health making susceptible to HIV, HSV-2, pathogenic microorganisms such as *Neisseria gonorrhoeae, Gardnerella vaginalis*, *Escherchia coli, Staphylococcus aureus, Peptostreptococcus anaerobius, P. bivia,* and *Chlamydia trachomatis*, pelvic inflammatory disease and pre-term delivery [6–13]. Therefore, Lactobacilli are considered gatekeepers of vaginal ecosystem and understanding their qualitative and quantitative diversity is an emphasized area of research [1].

The human microbiome project set out vaginal microenvironment as one of the priority site related to human health and disease and reported around 60 bacterial species that colonizes lower urinogenital tract of female dominated by *Lactobacillus crispatus, L. iners, L. gasseri, L. jensenii* [1, 14, 15]. Apparently, the diversity and dominance of *Lactobacillus* varies among different populations, ethnic groups, regions etc. Ravel et al [16] studied *Lactobacillus* composition in four ethnic groups and found that Asian and White women have higher vaginal *Lactobacillus* composition, dominated by *L. iners* and *L. crispatus* respectively, than Hispanic and Black women, who have higher proportion of strictly anaerobic bacteria and dominant *Lactobacillus* species is *L. iners*. In some other studies *Lactobacillus* composition has been further reported to vary among different populations and as such *L. iners, L. crispatus*, and *L. gasseri* were reported predominant among Chinese women [17], *L. acidophilus* among Mexicans and Swedish [18, 19], *L. crispatus*, *L. iners, L. gasseri, L. jenseni,* and *L. vaginalis* among South Africans [20]. A comparative study on Korean and Uganda women reported that *L. fermentum* was common in Korean, and *L. gasseri, L. reuteri, and L. vaginalis* in Ugandan women [21]. In India the biogeographical condition and population varies across different regions and as such the vaginal *Lactobacillus* species *L. iners*, *L. crispatus*, *L. reuteri*, *L. gasseri*, and *L. jensenii* have been found predominant in Mumbai (southwest region of India) [22], *L. reuteri, L. fermentum,* and *L. salivarius* in Delhi (central region of India) [23], *L. crispatus, L. gasseri,* and *L. jenseni* in Mysore (southern region of India) [24] and many of the regions still remain unexplored. A consensus of all studies explains four *Lactobacillus* species viz., *L. crispatus, L. jenseni, L. gasseri,* and *L. iners* [2] are predominant in vagina and among them *L. crispatus* and *L. jenseni* are considered most beneficial [25, 26] and their colonization corresponds to higher vaginal glycogen content [2].

*Lactobacillus* also restores vaginal health during pregnancy and its imbalance may cause BV and subsequent post-abortal infection, early and late miscarriage, histological chorioamnionitis, postpartum endometritis, preterm premature rupture of membranes and preterm birth [27–29]. In this context, a number of studies [30–35] had characterized vaginal microbiota during different stages of pregnancy in different biogeographical condition. However, such important studies are lacking from India.

Therefore, it is quite clear that the composition of *Lactobacillus* in vaginal ecosystem varies across population and dependent on many factors such as hormonal de-regulation, poor genital hygiene, improper vaginal douching, random sexual behaviour and in labouring women. Also different species of *Lactobacillus* offer different level of protection under different vaginal condition. Hence, structuring of vaginal microbiota in general and *Lactobacillus* in particular in different populations and their link with female health in normal and pregnant condition is highly appealing.

The present study evaluated the prevalence and the quantities of vaginal microbiota in the vagina of healthy fertile and pregnant women of Northeast India using culture-dependent techniques and subsequent 16S rRNA gene sequencing and analysis. Northeast India is genetically distinct from other parts of India and comprises of many tribes mainly of Austro-Asiatic and Tibeto-Burman background [36]. Further, they have unique dietary habits and lifestyle and these features in combination provide unique opportunity and challenges to decipher microbial composition in different habitats of human body [37] and we focussed on vaginal microbiota.

## Results

### Screening and characterization of samples, culture of bacterial isolates and sequencing

Vaginal swab samples were initially collected from 83 number of reproductive age females having age range 18–35. Among them 14 cases either had history of urinary tract infection, under antibiotic treatment, or has ongoing vaginal infection and excluded from the study (Additional file 1: Table S1). The remaining 69 samples according to age may be categorized as; 23 cases in age group 18–23, 25 cases in age group 24–29, and 21 cases in age group 30–35. The 69 samples further may be categorized as 29 non-pregnant and 40 pregnant cases and the pregnant cases may be categorized as; 12 first (I) trimester, 16 s (II) trimester, and 12 third ((III) trimester. The swab samples were streak plated in Nutrient Agar (NA), de Man, Rogosa and Sharpe (MRS), and Blood Agar (BA) media for microbial growth under ambient condition. While bacterial colonies appeared in NA and MRS media and a total of 154 bacterial isolates were obtained, no growth was observed in BA media. The number of isolates obtained from each sample, their gram staining and colony morphology, sample and colony code and corresponding sequencing code and accession numbers were detailed in Additional file 2: Table S2.

### Vaginal microbial diversity of females of Northeast India

The 16S rRNA gene based identification of the samples were based on a dual approach of homology search with database (Additional file 3: Table S3) and characteristic ML clustering (Additional file 6: Figure S1) of derived and their corresponding closely match database sequence. ML clustering was done with a total 270 sequences, that includes 154 derived and 116 database sequences (Additional file 4: Table S4), modelled by Kimura’s 2 parameter with gamma distributed rate across sites that describes best substitution pattern for the dataset according to Bayesian information criterion [38] (Additional file 5: Table S5). The homology search and ML clustering results are presented in a manageable form in Table 1.

**Table 1:** Identification of the bacterial isolates based on 16 s rRNA gene sequence

Sl. No.	Accession No.	No. of sequences derived	Species identified by homology search with database	Characteristic ML clustering as seen in Additional file 6: Figure S1
1	KU184494	1	*Bacillus subtilis*	Clustered distinct
2	KT906577 KT906579 KT906580	3	*Bifidobacterium breve*	Clustered distinct
3	KR264992	1	***Enterococcus avium***	The four species of *Enterococcus* genus clustered cohesively
4	KR265008 KT906587 KU184461	39	***Enterococcus faecalis***	
	KU184462 KU184464 KU184465			
	KU184460 KT906572 KU184469			
	KU184496 KU184470 KU184471			
	KU184472 KT906573 KU184498			
	KU184474 KT589112 KT589114			
	KT589115 KT589124 KR265004			
	KT589118 KT005519 KT005522			
	KT589119 KT589120 KT589122			
	KT589125 KT589126 KT589137			
	KT589136 KT589128 KT589105			
	KT589107 KT589108 KT589109			
	KT589110 KU184479 KT589111			
5	KP775931 KP775933	2	***Enterococcus faecium***	
6	KU184480 KR264993 KR264996	6	***Enterococcus hirae***	
	KU184476 KU184477 KU184478			
7	KT005520 KT589121	2	*Escherichia coli*	Clustered distinct
8	KP747671 KT597700 KP775922	3	*Janthinobacterium lividum*	Clustered distinct
9	KT906585	1	*Kocuria kristinae*	Clustered distinct
10	KT361204 KT589116 KT589117	5	*Lactobacillus brevis*	Clustered distinct
	KT589132 KT589133			
11	KU184473 KU184499 KP775929	10	*Lactobacillus fermentum*	Clustered distinct
	KT589130 KP775934 KP775923			
	KT589106 KR264983 KR264984			
	KR264986			
12	KP775927	1	*Lactobacillus gallinarum*	Clustered together with *Lactobacillus helviticus*
13	KT991842 KT991843 KU184463	6	*Lactobacillus gasseri*	Clustered distinct
	KU184466 KP775925 KR264987			
14	KT361211 KT361209 KP775926	3	*Lactobacillus helveticus*	Clustered together with *Lactobacillus gallinarum*
15	KU184500 KU184475	2	*Lactobacillus ingluviei*	Clustered distinct
16	KT906576 KT906578 KT906588	3	*Lactobacillus jensenii*	Clustered distinct
17	KT835013 KT835015 KT835018	25	Lactobacillus mucosae*	Clustered distinct and showed deep intraspecies divergence
	KT835007 KT835008 KT835009			
	KT835010 KT835011 KT835012			
	KT991844 KU184467 KU184468			
	KU184495 KU184497 KT597696			
	KU184483 KU184484 KU184485			
	KU184486 KU184487 KU184488			
	KU184489 KU184490 KU184491			
	KR264981			
18	KP775924	1	*Lactobacillus parafarraginis*	Clustered distinct
19	KR264991 KR264988	2	*Lactobacillus reuteri*	Clustered distinct
20	KT906581 KT906582 KT906583	6	*Propionibacterium avidum*	Clustered distinct
	KT906584 KT906571 KU184493			
21	KT906569	1	***Staphylococcus capitis***	The six species of Staphylococcus genus clustered cohesively
22	KR264994 KR264997 KR265000	3	***Staphylococcus caprae***	
23	KR265006 KT361206 KT361207	8	***Staphylococcus epidermidis***	
	KP747672 KT589123 KT589127			
	KT589131 KT589135			
24	KT005521	1	***Staphylococcus haemolyticus***	
25	KT589113 KP775928 KR264995	8	***Staphylococcus hominis***	
	KR264998 KR265001 KP775932			
	KR264999 KR264989			
26	KT906586	1	***Staphylococcus kloosii***	
27	KT906589 KT906574 KT906575	3	*Streptococcus agalactiae*	Clustered distinct
28	KT835014 KT906568 KT906570	3	*Streptococcus anginosus*	Clustered distinct
29	KT835016 KT835017	2	*Streptococcus gallolyticus*	Clustered distinct
30	KR264982	1	*Streptococcus infantarius*	Clustered distinct
31	KT361205	1	*Weissella confusa*	Clustered distinct

The Genbank accession number (column 2) of 16sRNA gene sequence of each of the 154 bacterial isolates and their corresponding similarity search result (column 4) (detailed in Additional file 3: Table S3) and characteristic ML clustering (column 5) (detailed in Additional file 6: Figure S1) is shown. Overall the 154 bacterial isolates were identified belonging to 31 species. *Enterococcus* and *Staphylococcus*, and two species of *Lactobacillus* (*L. gallinarum* and *L. helveticus*) showed cohesive intrageneric clustering in contrast to, cohesive cluster of all sequences of a particular species and distinct cluster with all other species sequences, and are shown here as bold. *Lactobacillus mucosae* showed deep intraspecies split and marked with asterisk (*)

Consensus match of derived sequences with a particular microbe in both the databases (NCBI and EZ-Taxon) in the range of similarity 97–100% and cohesive clustering of conspecific derived and database sequences confirmed straight forward identification of 80 sequences into 19 species. This includes *Bacillus subtilis, Bifidobacterium breve, Escherichia coli, Janthinobacterium lividum, Kocuria kristinae, Lactobacillus brevis, L. fermentum, L. gasseri, L. ingluviei, L. jensenii, L. mucosae, L. parafarraginis, L. reuteri, Propionibacterium avidum, Streptococcus agalactiae, S. anginosus, S. gallolyticus, S. infantarius,* and *Weissella confusa.* Among them *Lactobacillus mucosae* showed deep intraspecies split. One derived sequence (KP775927) that showed high similarity (98–99%) with *Lactobacillus gallinarum* and 3 sequences (KT361211, KT361209, and KP775926) that showed high similarity (97–99%) with *L. helveticus* based on homology search, clustered together with database sequences of *L. gallinarum* and *L. helveticus.* Similarly, 48 sequences identified as four species of *Enterococcus* (*E. avium, E. faecalis, E. faecium,* and *E. hirae*) based on homology search, and database sequences of the four *Enterococcus* species clustered together. Also, 22 sequences identified as 6 species of *Staphylococcus* (*S. capitis*, *S. caprae*, *S. epidermidis*, *S. haemolyticus*, *S. hominis*, and *S. kloosii*) and database sequences of the six *Staphylococcus* species clustered together.

In summary *Enterococcus faecalis* (25.3%) and *Lactbacillus mucosae* (16.2%) were found dominant species among the study population. 37.7% of the bacterial isolates were Lactobacilli*,* that includes 10 species, and their relative dominance is; *L. mucosae* (16.2%) *L. fermentum* (6.4%), *L. gasseri* (3.9%), *L. brevis* (3.2%), *L helveticus* and *L. jensenii* each 2%, *L. ingluviei* and *L. reuteri* each 1.3%, and *L. gallinarum* and *L. parafarraginis* each 0.6%. Clearly Lactobacilli percentage significantly varied among age group (*p* < 0.001) and we found in age group 18–23, 87% of females harbour *Lactobacillus*, in age group 24–29, 76% females harbour *Lactobacillus,* and in age group 30–35, 71.4% females harbour *Lactobacillus.*

### *Lactobacillus* composition in non-pregnant and pregnant women

**Fig. 1:**
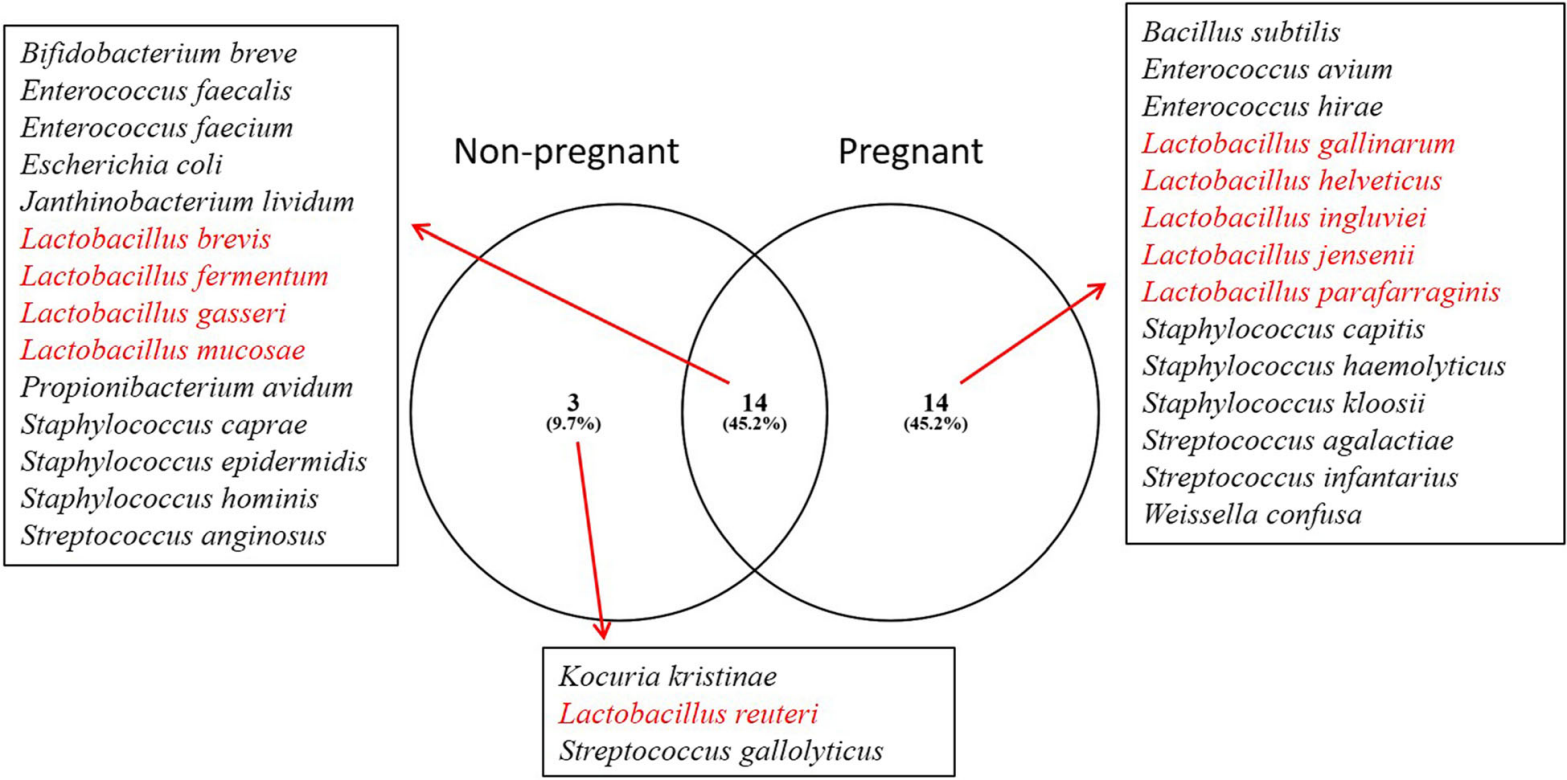
Bacterial colonization in non-pregnant and pregnant women. The numbers under each circle corresponds to number of species found under each condition and in parenthesis relative percentage shown. Clearly colonization is more in pregnant women than non-pregnant women

**Fig. 2:**
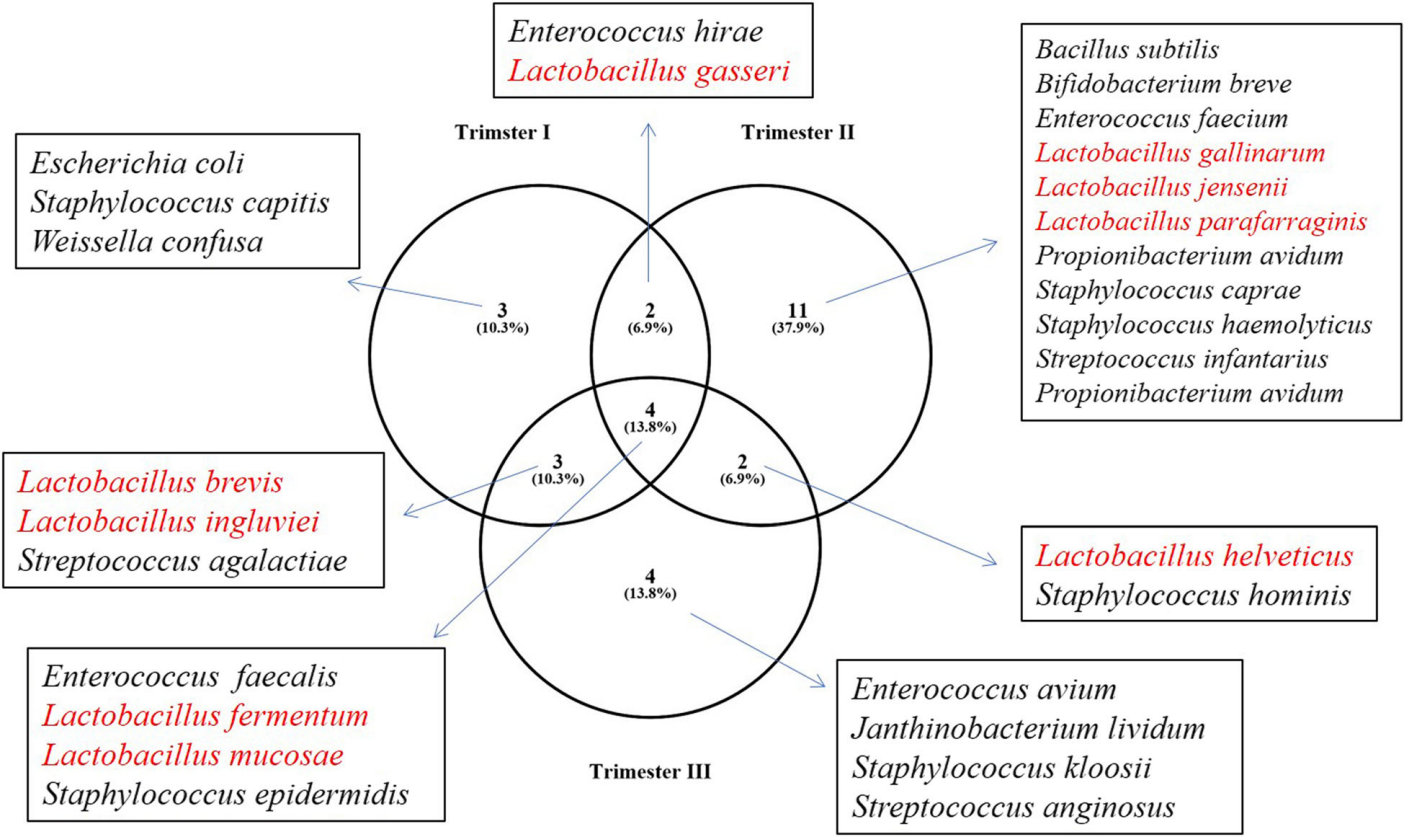
Bacterial colonization in three trimester of pregnant women. The numbers under each circle corresponds to number of species found under each trimester of pregnant women and in parenthesis relative percentage shown. Clearly bacterial colonization is higher in trimester II

One major objective of the present study is to categorize *Lactobacillus* diversity in non-pregnant and pregnant women and in three trimester of pregnant women. It is clearly evident in this study that the diversity of *Lactobacillus* increases in labouring women. As such we found only *L. reuteri* colonized non-pregnant women; 4 species *L. brevis, L. fermentum, L. gasseri*, and *L. mucosae* colonized both non-pregnant and pregnant women; and 5 species *L. gallinarum, L. helveticus, L. ingluviei, L. jensenii,* and *L. parafarraginis* colonized only pregnant women (Fig. 1). Trimester-wise the diversity of *Lactobacillus* is as follows: *L. fermentum* and *L. mucosae* overlapped in all three trimester, *L. brevis* and *L. ingluviei* overlapped in I and III trimester, *L. gasseri* overlapped in I and II trimester, *L. helveticus* overlapped in II and III trimester, while 3 species *L. gallinarum*, *L. jensenii*, and *L. parafarraginis* were found in only II trimester (Fig. 2). Again *Lactobacilli* percentage significantly varied among non-pregnant and pregnant women (*p* < 0.0002) and we found 58.6% of studied non-pregnant females harbour *Lactobacillus* while 90% of studied pregnant females harbour *Lactobacillus,* which may be further categorized as 83.3% trimester I*,* 93.8% trimester II, and 91.7% trimester III.

## Discussion

In women the vaginal ecosystem is dominated by Lactobacilli and this system has evolved to protect females health by inhibiting growth of pathogenic microbes. However, the characteristic microbial diversity varies across population [4, 16, 39–42]. Women in different settings face different geographical condition, dietary habits, and lifestyle. In particular, peoples of Northeast India are from Austro-Asiatic and Tibeto-Burman background and are genetically different from other parts of India [36, 43, 44]. Therefore, it is tempting to screen and compare how the vaginal microbiota is structured in females in Northeast India and how it differs from other population.

In this study altogether we isolated 154 bacterial isolates in NA and MRS media from 69 females; which includes 40 pregnant and 29 non-pregnant cases. BA was used as a selective media for Lactobacilli, especially *Lactobacillus iners,* however, no colonies appeared in BA. Previous studies [45–47] advocates that *L. iners* grows in transitional phase of BV and healthy state, which is not the case in this study, and this tentatively is the reason for no growth in BV. However, we believe a detail sampling including healthy and BV cases in future studies may provide a comprehensive insight on *Lactobacillus* diversity among the females of Northeast India.

A dual approach of homology search and ML clustering confirmed the 154 bacterial isolates belonging to 31 species and 37.7% of the bacterial isolates are *Lactobacillus*. Interestingly, we found *L. mucosae* is the dominant species and *L. fermentum, L. gasseri, L. brevis, L. helveticus, L. jensenii, L. ingluviei, L. reuteri, L. gallinarum*, and *L. parafarraginis* together forms the Lactobacilli complex that colonizes vaginal microenvironment among the females of Northeast India. This is the first report of its type from Northeast India and has implications for global comparison of Lactobacilli composition. Clearly, vaginal *Lactobacillus* composition among Northeast India population is different from other parts of India [22–24] and various regions/countries of the World [16–21]. *Lactobacillus mucosae* has been reported as less common vaginal microflora in previous studies [41, 48], however dominance of *L. mucosae* than *L. crispatus* and *L. jensenii* (as observed in most of the previous studies [2, 16]) observed in this study in Northeast Indian females is an intriguing finding and tentatively reflects different vaginal set-up. In particular, different *Lactobacillus* species possess unique repertoire of protein families which helps in specific community adaptations [49, 50]. *Lactobacillus mucosae* has been reported to express Lam29 protein in intestinal epithelial cells, that significantly improves its adhesion for human blood group A and B antigens and out competes pathogens to protect intestinal mucosa [51]. In human vagina, the epithelial layer similarly acts as a barrier for mucosal layer, which the pathogen wants to penetrate and *Lactobacillus* wants to protect by adhesion and outcompeting pathogens [52–55]. However, this adhesion is dependent on genetic repertoire of both the host and the bacteria [49, 50]. Further, the adhesion and colonization of *Lactobacillus* is also dependent on hormonal level (especially estrogen), glycogen content of epithelial cell, and availability of nutrients [3]. We hypothesize *Lactobacillus mucosae* outcompetes other organisms for receptors and nutrients in the vaginal epithelial cells of females of Northeast India and this claim needs more insight from future studies. Nevertheless, this finding is also tempting to explore probiotic potential of *L. mucosae* than *L. crispatus* and *L. jensenii* among women of Northeast India and correlation with estrogen level and deposition of glycogen in vaginal epithelium.

Besides *Lactobacillus* that constituted 37.7% of bacterial isolates, we also found 10 other lactic acid bacteria species (39.61%) viz., *Bifidobacterium breve, Enterococcus avium, E. faecalis, E. faecium, E. hirae, Streptococcus agalactiae, S. anginosus, S. gallolyticus, S. infantarius,* and *Weissella confusa* with predominance of *Enterococcus faecalis* (25.3%). Among them *S. infantarius, S. gallolyticus, S. agalactiae, S. anginosus, E. avium, E. hirae* are opportunistic pathogens and causes severe invasive infections [56–60].

The study also reports 4 species of *Enterococcus* viz., *E. avium, E. faecalis, E. faecium,* and *E. hirae*, 6 species of *Staphylococcus* (14.28%) viz., *S. capitis*, *S. caprae*, *S. epidermidis*, *S. haemolyticus*, *S. hominis*, and *S. kloosii* and 2 species of *Lactobacillus* viz., *L. gallinarum*, and *L. helveticus* with cohesive congeneric clustering. This clearly reveals congeneric exchange of 16S rRNA gene segment, may be due to horizontal gene transfer or homologous recombination, which is common among microbes [61]. These key findings together with the observation of deep intraspecies split in *Lactobacillus mucosae* warrants genome-wide study of *Enterococcus*, *Staphylococcus* and *Lactobacillus* in future studies to understand intraspecies genomic variability, possible emergence of new strains/variants and its impact on female health of Northeast India. Nevertheless, *Propionibacterium avidum* (3.90%)*, Bacillus subtilis, Escherchia coli, Janthinobacterium lividum,* and *Kocuria kristinae* (each 0.64%) represented renaming of the microbiota found in this study. *Lactobacillus ingluviei* and *L. parafarraginis* found in this study have never been previously reported from human vagina [62, 63] and so this is the first case of their report from human vagina.

The vaginal microbiota have a major impact during different stages of pregnancy [30] and therefore we assessed how the microbes, and *Lactobacillus* in particular, are structured among non-pregnant and different stages of pregnant women. We found *Lactobacillus* percentage is highest in the age group 18–23 (87%) and successively decreases (71–76%). This finding is consistent with previous studies [64] and therefore as female attains age and *Lactobacillus* composition decreases, the pathogenic microbes have higher chance of colonization and thus extra precaution must be followed. In particular, aged women may be supplemented with *Lactobacillus* probiotic, which is predominant in their population, since it is observed herein and some previous studies [4, 16, 39–42] that predominance of *Lactobacillus* species varies across population. The *Lactobacillus* composition in pregnant women showed a sharp increase (90%) compared to non-pregnant women (58.6%) similar to earlier study [65] and this perhaps is the best way to protect vaginal health in labour periods. We further evidenced higher colonization of Lactobacilli in trimester II followed by trimester III and I. These features must be explored in more details for specific functional characterization of different species of *Lactobacillus* in different stages of pregnancy.

## Conclusion

The study clearly highlighted importance of assessment of vaginal microbiota in different populations, groups, ethnics, religions etc. The vaginal microbial composition studied herein revealed some interesting new information’s such as: i) *Lactobacillus mucosae* rather than *L. crispatus* or *L. iners* is dominant among females of Northeast India and this may be related to *Lacotbacillus* adaptation in particular population mainly due to genetic variability, ii) deep intraspecies split among *L. mucosae* revealed population level divergence and may be due to evolution of various strains active under specific conditions, iii) Lactobacilli percentage among Northeast India females is less and tentatively compensated by other LABs, iv) parallel to *L. mucosae*, *Enterococcus faecalis* is also dominant among females of Northeast India and its specific role must be explored. The Lactobacilli composition in non-pregnant and pregnant cases is consistent to previous studies however specific trimester-wise colonization of particular *Lactobacillus* species needs to be explored in more details. This study is the first report of its type from Northeast India and although we used culture-dependent technique, we identified around 31 vaginal microbial species and importantly revealed unique Lactobacilli diversity and dominance. We believe our study provided an interesting clue on vaginal *Lactobacillus* composition. This is tempting for future studies to include broad spectrum culture independent technique together with culture dependent technique for better understanding deficiency and dominance of particular vaginal *Lactobacillus* species and its relation to female health of Northeast India; Northeast India females have high rate of cervical cancer and Lactobacilli have major role in acquisition and persistence of Human Papilloma Virus [66] and regulation of cervical cancer [67].

## Methods

### Sample collection and culture of microbes

Vaginal swab samples were collected from 83 numbers of healthy pregnant and non-pregnant women during the period from 23 July 2012 to 29 November 2015 (Additional file 1: Table S1). A conscious effort was made to collect samples from volunteers who did not have any apparent vaginal infection. The present experiment excluded the volunteers who had antibiotic treatment in last one and half months, and who has history of urinary tract infection in last 6 months, any homogenous white discharge from vagina, and whose pH higher than 4.5, and who have the problem of itching, bleeding from vagina prior to sampling. All women were at reproductive age i.e. 18–35. Necessary clearance from the Institutional Ethical Committee, Karimganj College (KC/IEC/2012/M-1/10, dated: 23 Jun 2012) before the commencement of the study was obtained. The samples were collected aseptically by a practicing gynaecologist of the Red Cross Society of India, Karimganj, Assam (India). Initially, the experimental procedure was explained to the volunteers and when they agreed to participate in the study, they were asked to sign a written consent which was prepared as per the recommendation of the Institutional Ethical Committee. The collection of the samples was made after obtaining formal consent. A sterile swab (Hi Media Laboratory Pvt. Ltd., India) was inserted into the vaginal lumen and the samples were collected from the posterior fornix of vagina. The swabs were collected separately in sterile tubes. The swabs were used within 3 h of collection. The microbes from the swabs were transferred to sterile NA, MRS Agar, and BA media by streak-plate method. It is to be mentioned that while MRS and BA are selective media for Lactobacilli, NA was used as a general starter media to get a general view on microbes present apart from *Lactobacillus* in vaginal microenvironment of females of NE India. The plates were incubated for 48 h at 37 °C both under aerobic and anaerobic condition to allow the growth of microbial colonies. During collection of samples a statistical approach called convenience method/sampling was applied [68] (Additional file 1: Table S1).

### DNA isolation and PCR

The microbial genomic DNA was isolated following the protocol as mentioned in our previous paper [69]. The amplification of the 16S rRNA gene was done using published universal primers 27F Forward primer (AGAGTTTGATCMTGGCTCAG) and 1492R Reverse primer (5ˈ-GGTTACCTTGTTACGACTT-3ˈ) [70]. The 10 μl PCR reaction mixture contained template DNA 1 μl, 20 picomole of each primer, Master Mix 2X (HiMedia, India) 5 μl, and nuclease-free water 2 μl. The PCR thermal condition includes initial denaturation at 94 °C for 2 min, 35 cycles of 94 °C for 30 s, 49.3 °C for 30 s, 72 °C for 1 min and final extension at 72 °C for 3 min in S1100 thermal cycler (BioRad, USA). The PCR products were visualized in 1% agarose gel and images were captured by a Gel DOC machine (BioRad, USA).

### 16S rRNA gene sequencing

The 1.5 Kb amplicons were sequenced using the facilities provided by the MERK, Bangalore, India and Xcelris, Hyderabad, India. After obtaining the sequencing data, these were analysed and edited using Sequence scanner software v1.0 [57] (Applied Biosystem Inc., CA, USA). Alignments are prepared by ClustalX [71] and DNA contigs. Thereafter, the sequence was matched using BLASTn (NCBI database) [72] to identify its closest relatives. The sequences were submitted in the NCBI database using the submission tool BankIt and the accession numbers were obtained.

### Identification of the microbes

For Identification of the microbes we followed a dual approach of sequence based homology search and clustering (Additional file 7: Figure S2). For homology search we compared a particular sequence for similarity with two separate databases NCBI using BLASTn approach [72] and EZ-Taxon (www.eztaxon.org/). For clustering we used Maximum Likelihood method [73] structured by Kimura’s 2 parameter [57] model and the computation platform used was MEGAX [74]. The high similarity (97–100%) of a sequence with database sequences of a particular microbe coupled with cohesive clustering of developed and database sequence of the particular microbe and distinct cluster with respect of other developed and database congeners sequences confirmed identification of the microbe with high precision.

The Lactobacilli percentage across different age group and pregnant and non-pregnant women were compared using Student’s t-test.

## Additional files

Additional file 1: **Table S1.** Sample collection information. (DOCX 28 kb)

Additional file 2: **Table S2.** Cell morphology, Gram staining of the bacterial isolates and their corresponding GenBank accession numbers of 16S rRNA gene sequence. (DOCX 36 kb)

Additional file 3: **Table S3.** Homology search of the sequences in database. (DOCX 38 kb)

Additional file 4: **Table S4.** Developed and database sequences. (XLSX 13 kb)

Additional file 5: **Table S5.** Model test result. (XLS 49 kb)

Additional file 6: **Figure S1.** Maximum Likelihood based clustering of developed and database sequences. The sequences of same species clustered together and distinct with respect to sequences of other species. (PDF 50 kb)

Additional file 7: **Figure S2.** 16S rRNA gene based identification of the microbes is based on dual approach of homology search and ML clustering. To confirm identification we considered consensus match of our query sequence with a particular microbe on both the database and cohesive clustering of developed and database sequence of the particular microbe (here shown as Species A and Species B) and distinct with respect to other microbes (all species A members clustered distinct with all species B members). (JPG 250 kb)

## Data Availability

All the sequences used in this study have been submitted to NCBI and the details can be checked using their respective accession numbers (please see Additional file 2: Table S2) in NCBI (https://www.ncbi.nlm.nih.gov/).
