# Supplementary material for: Contrasting diversity of vaginal lactobacilli among the females of Northeast India
Source: BMC Microbiol. 2019 Aug 27;19:198. doi: 10.1186/s12866-019-1568-6 (PMC6712660; doi:10.1186/s12866-019-1568-6)
Supplement: Supplementary file 1 — Table S1. Sample collection information. (DOCX 28 kb) [file 12866_2019_1568_MOESM1_ESM.docx]

**Table S1:** Sample collection information.

| Sl No. | Sample code | Age group | Pregnancy  status | Pregnancy  trimester | Remark |
| --- | --- | --- | --- | --- | --- |
|  | W3 | 18-23 | + | I |  |
|  | W6 |  |  |  | Did not met study criteria |
|  | W9 |  | + | II |  |
|  | W12 |  |  |  | Did not met study criteria |
|  | W14 |  |  |  | Did not met study criteria |
|  | W17 |  | + | III |  |
|  | W18 |  |  |  | Did not met study criteria |
|  | W19 |  | + | II |  |
|  | W20 |  | + | II |  |
|  | W24 |  |  |  | Did not met study criteria |
|  | W30 |  |  |  | Did not met study criteria |
|  | W31 |  | - |  |  |
|  | W33 |  | + | I |  |
|  | W34 |  | + | II |  |
|  | W38 |  | + | II |  |
|  | W40 |  | - |  |  |
|  | W41 |  | + | I |  |
|  | W45 |  | + | II |  |
|  | W46 |  |  |  | Did not met study criteria |
|  | W47 |  | + | II |  |
|  | W49 |  |  |  | Did not met study criteria |
|  | W50 |  | - |  |  |
|  | W53 |  | - |  |  |
|  | W55 |  | - |  |  |
|  | W57 |  | - |  |  |
|  | W62 |  | + | III |  |
|  | W64 |  |  |  | Did not met study criteria |
|  | W66 |  | - |  |  |
|  | W70 |  | - |  |  |
|  | W73 |  | + | I |  |
|  | W74 |  | + | III |  |
|  | W1 | 24-29 | + | II |  |
|  | W5 |  | + | I |  |
|  | W7 |  | - |  |  |
|  | W8 |  |  |  | Did not met study criteria |
|  | W10 |  | - |  |  |
|  | W13 |  | + | I |  |
|  | W15 |  | - |  |  |
|  | W16 |  | + | II |  |
|  | W22 |  | - |  |  |
|  | W25 |  | + | III |  |
|  | W26 |  | + | II |  |
|  | W28 |  | + | II |  |
|  | W35 |  | - |  |  |
|  | W37 |  | + | III |  |
|  | W43 |  | + | III |  |
|  | W44 |  | - |  |  |
|  | W48 |  | + | II |  |
|  | W51 |  | + | II |  |
|  | W52 |  | - |  |  |
|  | W56 |  | + | II |  |
|  | W60 |  | - |  |  |
|  | W65 |  | - |  |  |
|  | W69 |  | + | I |  |
|  | W72 |  | + | III |  |
|  | W76 |  |  |  | Did not met study criteria |
|  | W79 |  | + | III |  |
|  | W81 |  | + | II |  |
|  | W82 |  | + | I |  |
|  | W2 | 30-35 |  |  | Did not met study criteria |
|  | W4 |  | + | II |  |
|  | W11 |  | - |  |  |
|  | W21 |  |  |  | Did not met study criteria |
|  | W23 |  | + | III |  |
|  | W27 |  | - |  |  |
|  | W29 |  | - |  |  |
|  | W32 |  | + | I |  |
|  | W36 |  | - |  |  |
|  | W39 |  | + | III |  |
|  | W42 |  | - |  |  |
|  | W54 |  | + | III |  |
|  | W59 |  | - |  |  |
|  | W59 |  |  |  | Did not met study criteria |
|  | W61 |  | - |  |  |
|  | W63 |  | + | III |  |
|  | W67 |  | + | III |  |
|  | W68 |  | - |  |  |
|  | W71 |  | - |  |  |
|  | W75 |  | - |  |  |
|  | W77 |  | + | I |  |
|  | W78 |  | - |  |  |
|  | W80 |  | + | II |  |
|  | W83 |  | + | I |  |
