# Supplementary material for: Contrasting diversity of vaginal lactobacilli among the females of Northeast India
Source: BMC Microbiol. 2019 Aug 27;19:198. doi: 10.1186/s12866-019-1568-6 (PMC6712660; doi:10.1186/s12866-019-1568-6)
Supplement: Supplementary file 2 — Table S2. Cell morphology, Gram staining of the bacterial isolates and their corresponding GenBank accession numbers of 16S rRNA gene sequence. (DOCX 36 kb) [file 12866_2019_1568_MOESM2_ESM.docx]

**Table S2:** Cell morphology, Gram staining of the bacterial isolates and their corresponding GenBank accession numbers of 16S rRNA gene sequence.

| Sl No. | Sample code | Colony | Gram  staining | Colony  morphology | Sample code  For sequencing | Accession no. |
| --- | --- | --- | --- | --- | --- | --- |
| 1 | W1 | K531 | + | Large sized, creamy white | W1-K531 | KT589119 |
| 2 |  | A6 | + | Very Small, white | W1-A6 | KT835009 |
| 3 |  | K7 | + | Very Small, white | W1-K7 | KP775924 |
| 4 | W3 | K80 | + | Very Small, white | W3-K80 | KT589106 |
| 5 |  | K533 | - | Small, creamy white | W3-K533 | KT005520 |
| 6 |  | AN67 | + | Large sized, creamy white | W3-AN67 | KU184470 |
| 7 | W4 | L129 | + | Small, creamy white | W4-L129 | KU184487 |
| 8 |  | K534 | + | Medium sized, white colony | W4-K534 | KT589120 |
| 9 | W5 | K76 | + | Small, white colony | W5-K76 | KR264981 |
| 10 |  | K536 | + | Medium sized, white colony | W5-536 | KT589122 |
| 11 |  | L3 | + | Very Small, white | W5-L3 | KP747672 |
| 12 | W7 | AN11 | + | Large, white colony | W7-AN11 | KT906577 |
| 13 |  | AN4 | + | Very small, white | W7-AN4 | KT906571 |
| 14 | W9 | A5 | + | Small, white colony | W9-A5 | KT835008 |
| 15 |  | K538 | + | Medium sized, creamy white colony | W9-K538 | KT589124 |
| 16 | W10 | K121 | + | Small, white colony | W10-K121 | KT589130 |
| 17 |  | K539 | + | Small, white colony | W10-K539 | KT589125 |
| 18 |  | KA114 | + | Small, white colony | W10-KA114 | KR265006 |
| 19 | W11 | AN54 | + | Small, white | W11-AN54 | KT991844 |
| 20 |  | K540 | + | Small, yellowish | W11-K540 | KT589126 |
| 21 |  | K17 | + | Very small, white | W11-K17 | KT361206 |
| 22 | W13 | A27 | + | Very small, white | W13-A27 | KU184494 |
| 23 |  | AN62 | + | Large, white colony | W13-AN62 | KU184469 |
| 24 |  | AN70 | + | Large, white colony | W13-AN70 | KU184473 |
| 25 | W15 | K12 | + | Very small, white | W15-K12 | KP775929 |
| 26 |  | K545 | + | Medium, white | W15-K545 | KT589128 |
| 27 | W16 | AN12 | + | Small, white | W16-AN12 | KT906578 |
| 28 |  | K18 | + | Small, yellowish | W16-K18 | KT361207 |
| 29 | W17 | L17 | + | Small, white | W17-L17 | KP775934 |
| 30 |  | K544 | + | Very large, creamy white | W17-K544 | KT589136 |
| 31 |  | K20 | - | Medium, Yellowish | W17-K20 | KT597700 |
| 32 | W19 | K10 | + | Medium, Yellowish | W19-K10 | KP775927 |
| 33 |  | K543 | + | Medium, Yellowish | W19-K543 | KT589137 |
| 34 | W20 | K3 | + | Very Small, white | W20-K3 | KT361209 |
| 35 |  | K14 | + | Large, white | W20-K14 | KP775931 |
| 36 |  | K79 | + | Small, white | W20-K79 | KR264982 |
| 37 | W22 | K16 | + | Large, white | W22-K16 | KP775933 |
| 38 |  | K107 | + | Small, white | W22-K107 | KR264997 |
| 39 | W23 | K81 | + | Small, white | W23-K81 | KR264984 |
| 40 | W25 | K9 | + | Small, white | W25-K9 | KP775926 |
| 41 |  | AN6 | + | Large, white | W25-AN6 | KT906572 |
| 42 | W26 | AN18 | + | Small, white | W26-AN18 | KT906581 |
| 43 | W27 | K124 | + | Small, white | W27-124 | KT597696 |
| 44 |  | AN7 | + | Large, white | W27-AN7 | KT906573 |
| 45 | W28 | AN10 | + | Small, white | W28-AN10 | KT906576 |
| 46 |  | K92 | + | Large, white | W28- K92 | KU184479 |
| 47 |  | AN25 |  | Medium, white | W29- AN25 | KT906585 |
| 48 | W29 | AN19 | + | Large, white | W29- AN19 | KT906582 |
| 49 |  | AN5 | + | Large, white | W31-AN5 | KU184460 |
| 50 | W32 | A4 | + | Small, white | W32-A4 | KT835007 |
| 51 |  | K75 | + | Medium sized, white colony | W32-K75 | KT361205 |
| 52 | W33 | A7 | + | Small, white | W33-A7 | KT835010 |
| 53 |  | K122 | + | Small, white | W33-K122 | KT589132 |
| 54 |  | AN42 | + | Large, white | W33-AN42 | KU184461 |
| 55 | W34 | K85 | + | Small, white | W34-K85 | KR264986 |
| 56 |  | AN13 | + | Large, white | W34-AN13 | KT906579 |
| 57 | W35 | AN20 | + | Medium , white | W35-AN20 | KT906583 |
| 58 | W36 | K105A | + | Medium, yellowish | W36-K105A | KR264995 |
| 59 |  | AN44 | + | Large, white | W36-AN44 | KU184462 |
| 60 |  | K548 | + | Medium, creamy | W36-K548 | KT589135 |
| 61 | W37 | K120 | + | Small, white | W37-K120 | KT361211 |
| 62 |  | K123 | + | Large, white | W37-K123 | KT589133 |
| 63 |  | AN47 | + | Large, white | W37-AN47 | KU184464 |
| 64 | W38 | AN49 | + | Large, white | W38-AN49 | KU184465 |
| 65 |  | AN31 | + | Small, white | W38-AN31 | KT906588 |
| 66 |  | AN23 | + | Medium, white | W38-AN23 | KU184493 |
| 67 | W39 | K79 | + | Small, white | W39-K79 | KU184500 |
| 68 |  | KA115 | + | Small, white | W39-KA115 | KT361204 |
| 69 | W40 | A15 | + | Small, white | W40-A15 | KT835016 |
| 70 | W41 | AN88 | + | Small, white | W41-AN79 | KU184475 |
| 71 |  | K84 | + | Large, white | W41-K84 | KT589108 |
| 72 |  | K537 | + | Large, white | W41-K537 | KT589123 |
| 73 | W42 | K6 | + | Small, white | W42-K6 | KP775923 |
| 74 |  | K89 | + | Large, white | W42-K89 | KT589109 |
| 75 | W43 | K132 | + | Small, white | W43-K132 | KU184490 |
| 76 |  | K91 | + | Large, white | W43-K91 | KT589110 |
| 77 |  | K541 | + | Large, white | W43-K541 | KT589127 |
| 78 | W44 | K535 | - | Medium, white | W44-K535 | KT589121 |
| 79 |  | K547 | + | Large, creamy | W44-K547 | KT589131 |
| 80 | W45 | L80 | + | Small, white | W45-L80 | KR264983 |
| 81 |  | K86 | + | Medium, white | W45-K86 | KU184478 |
| 82 |  | AN24 | + | Medium, white | W45-AN24 | KT906584 |
| 83 | W47 | K131 | + | Small, white | W47-K131 | KU184489 |
| 84 |  | K93 | + | Large, white | W47-K93 | KT589111 |
| 85 |  | L102 | + | Medium, white | W47-L102 | KU184480 |
| 86 | W48 | K133 | + | Small, white | W48-K133 | KU184491 |
| 87 |  | K102 | + | Large, white | W48-K102 | KT589112 |
| 88 |  | L527 | + | Large, yellowish | W48-L527 | KT005521 |
| 89 | W50 | K87 | + | Small, white | W50-K87 | KR264987 |
| 90 |  | K97 | + | Small, white | W50-K97 | KR264988 |
| 91 |  | AN68 | + | Large, white | W50-AN68 | KU184471 |
| 92 | W51 | AN58 | + | Small, white | W51-AN58 | KU184468 |
| 93 |  | K70 | + | Medium, white | W51-K70 | KU184477 |
| 94 | W52 | AN66 | + | Small, white | W52-AN66 | KU184497 |
| 95 | W53 | K105 | + | Medium, white | W53-K105 | KR264994 |
| 96 | W54 | A9 | + | Small, white | W54-A9 | KT835012 |
| 97 |  | K108 | + | Large, white | W54-K108 | KT589114 |
| 98 |  | KA105 | + | Medium, white | W54-KA105 | KT589113 |
| 99 | W55 | K113 | + | Small, white | W55-K113 | KT589117 |
| 100 |  | K111 | + | Medium, white | W55-K111 | KT589115 |
| 101 | W56 | A113 | + | Small, white | W56-A13 | KT835015 |
| 102 |  | K69 | + | Medium, white | W56-K69 | KU184476 |
| 103 |  | K109 | + | Medium, white | W56-K109 | KR265000 |
| 104 |  | K130 | + | Small, white | W56-K130 | KU184488 |
| 105 | W57 | K8 | + | Small, white | W57-K8 | KP775925 |
| 106 |  | A20 | + | Large, white | W57-A20 | KT835018 |
| 107 |  | K115 | + | Large, white | W57-K115 | KT589118 |
| 108 | W58 | A10 | + | Small, white | W58-A10 | KT835013 |
| 109 |  | AN3 | + | Small, yellowish | W58-AN3 | KT906570 |
| 110 | W60 | AN69 | + | Large, white | W60-AN69 | KU184472 |
| 111 |  | AN12 | + | Small, yellowish | W60-AN12 | KT835014 |
| 112 | W61 | AN14 | + | Small, white | W61-AN14 | KT906580 |
| 113 |  | K5 | + | Medium, yellowish | W61-K5 | KP775922 |
| 114 | W62 | AN74 | + | Small, white | W62-AN74 | KU184499 |
| 115 | W63 | AN59 | + | Small, white | W63-AN59 | KU184495 |
| 116 |  | AN1 | + | Medium, white | W63-AN1 | KT906568 |
| 117 | W65 | K112 | + | Small, white | W65-K112 | KT589116 |
| 118 |  | AN78 | + | Large, white | W65-AN78 | KU184474 |
| 119 | W66 | K100 | + | Small, white | W66-K100 | KR264991 |
| 120 |  | K2 | - | Medium, yellowish | W66-K2 | KP747671 |
| 121 | W67 | A8 | + | Small, white | W67-A8 | KT835011 |
| 122 |  | AN22 | + | Small, yellowish | W67-AN22 | KT906589 |
| 123 | W68 | KA107 | + | Medium white | W68-KA107 | KR264998 |
| 124 | W69 | AN64 | + | Large, white | W69-AN64 | KU184496 |
| 125 | W70 | AN72 | + | Large, white | W70-AN72 | KU184498 |
| 126 |  | A16 | + | Small, white | W70-A16 | KT835017 |
| 127 |  | K14 | + | Medium, white | W71-K14 | KP775932 |
| 128 | W72 | K83 | + | Large, white | W72-K83 | KT589107 |
| 129 |  | KA109 | + | Medium, white | W72-KA109 | KR265001 |
| 130 | W73 | K77 | + | Large, white | W73-K77 | KT589105 |
| 131 |  | AN57 | + | Small, white | W73-AN57 | KU184467 |
| 132 |  | KA104 | + | Medium, white | W73-KA104 | KR264993 |
| 133 | W74 | L125 | + | Small, white | W74-L125 | KU184483 |
| 134 |  | K104 | + | Medium white | W74-K104 | KR264992 |
| 135 |  | AN26 | + | Medium, white | W74-AN26 | KT906586 |
| 136 | W75 | AN35 | + | Small, white | W75-AN35 | KT991842 |
| 137 |  | KA108 | + | Medium, white | W75-KA108 | KR264999 |
| 138 | W77 | AN36 | + | Small, white | W77-AN36 | KT991843 |
| 139 |  | K530 | + | Large, white | W77-K530 | KT005522 |
| 140 |  | KA106 | + | Medium, white | W77-KA106 | KR264996 |
| 141 | W78 | K126 | + | Small, white | W78-K126 | KU184484 |
| 142 |  | K528 | + | Large, white | W78-K528 | KT005519 |
| 143 | W79 | AN9 | + | Medium, white | W79-AN9 | KT906575 |
| 144 | W80 | AN46 | + | Small, white | W80-AN46 | KU184463 |
| 145 |  | KA117 | + | Large, white | W80-K117 | KR265008 |
| 146 |  | K11 | + | Medium, white | W80-K11 | KP775928 |
| 147 | W81 | AN50 | + | Small, white | W81-AN50 | KU184466 |
| 148 |  | K98 | + | Medium, white | W81-K98 | KR264989 |
| 149 | W82 | K127 | + | Small, white | W82-K127 | KU184485 |
| 150 |  | KA111 | + | Large, white | W82-KA111 | KR265004 |
| 151 |  | AN2 | + | Large, white | W82-AN2 | KT906569 |
| 152 | W83 | K128 | + | Small, white | W83-K128 | KU184486 |
| 153 |  | AN30 | + | Large, white | W83-AN30 | KT906587 |
| 154 |  | AN8 | + | Medium white | W83-AN8 | KT906574 |
