# Supplementary material for: Contrasting diversity of vaginal lactobacilli among the females of Northeast India
Source: BMC Microbiol. 2019 Aug 27;19:198. doi: 10.1186/s12866-019-1568-6 (PMC6712660; doi:10.1186/s12866-019-1568-6)
Supplement: Supplementary file 3 — Table S3. Homology search of the sequences in database. (DOCX 38 kb) [file 12866_2019_1568_MOESM3_ESM.docx]

**Table S3:** Homology search of the sequences in database.

| Sl No. | Sample code | Accession  no. | Homology search at BLASTN | | Homology search at bacterial database | |
| --- | --- | --- | --- | --- | --- | --- |
|  |  |  | Name of the organism | % similarity | Name of the organism | % similarity |
| 1 | W80-K117 | KR265008 | *Enterococcus faecalis (HM776178)* | 99 | *Enterococcus faecalis (*[*ASDA01000001*](https://www.ezbiocloud.net/16SrRNA?ac=ASDA01000001)*)* | 99.62 |
| 2 | W10-KA114 | KR265006 | *Staphylococcus epidermidis (KX760138)* | 99 | *Staphylococcus epidermidis (*[*L37605*](https://www.ezbiocloud.net/16SrRNA?ac=L37605)*)* | 99.59 |
| 3 | W82-KA111 | KR265004 | *Enterococcus faecalis (MH179309)* | 99 | *Enterococcus faecalis (*[*ASDA01000001*](https://www.ezbiocloud.net/16SrRNA?ac=ASDA01000001)*)* | 100 |
| 4 | W72-KA109 | KR265001 | *Staphylococcus hominis (MH017408)* | 100 | *Staphylococcus hominis subsp. hominis*  *(*[*X66101*](https://www.ezbiocloud.net/16SrRNA?ac=X66101)*)* | 99.76 |
| 5 | W56-K109 | KR265000 | *Staphylococcus caprae ( KU922215)* | 99 | *Staphylococcus caprae (*[*L37599*](https://www.ezbiocloud.net/16SrRNA?ac=L37599)*)* | 99.47 |
| 6 | W75-KA108 | KR264999 | *Staphylococcus hominis*  *(MG685880)* | 99 | *Staphylococcus hominis subsp. novobiosepticus*  *(*[*AB233326*](https://www.ezbiocloud.net/16SrRNA?ac=AB233326)*)* | 99.62 |
| 7 | W68-KA107 | KR264998 | *Staphylococcus hominis (MH017408)* | 99 | *Staphylococcus hominis subsp. hominis (*[*AB233326*](https://www.ezbiocloud.net/16SrRNA?ac=AB233326)*)* | 99.72 |
| 8 | W22-K107 | KR264997 | *Staphylococcus caprae (KX946138)* | 99 | *Staphylococcus caprae*  *(AB009935)* | 99.67 |
| 9 | W77-KA106 | KR264996 | *Enterococcus hirae (KT261200)* | 100 | *Enterococcus hirae*  *(CP003504)* | 100 |
| 10 | W53-K105 | KR264994 | *Staphylococcus caprae (KX946138)* | 100 | *Staphylococcus caprae*  *(L37599)* | 99.59 |
| 11 | W73-KA104 | KR264993 | *Enterococcus hirae (MF327681)* | 100 | *Enterococcus hirae (CP003504)* | 99.83 |
| 12 | W74-K104 | KR264992 | *Enterococcus avium (KE136366)* | 100 | *Enterococcus avium (MH111604)* | 99 |
| 13 | W66-K100 | KR264991 | *Lactobacillus reuteri (LC369122)* | 99 | *Lactobacillus reuteri (AP007281)* | 99.61 |
| 14 | W81-K98 | KR264989 | *Staphylococcus hominis subsp. novobiosepticus*  *(MG201782)* | 99 | *Staphylococcus hominis subsp. novobiosepticus (AB233326)* | 100 |
| 15 | W50-K97 | KR264988 | *Lactobacillus reuteri (KX688655)* | 99 | *Lactobacillus reuteri (AP007281)* | 99 |
| 16 | W50-K87 | KR264987 | *Lactobacillus gasseri (MH113149)* | 99 | *Lactobacillus gasseri (CP000413)* | 98.92 |
| 17 | W34-K85 | KR264986 | *Lactobacillus fermentum*  *(MH041171)* | 100 | *Lactobacillus fermentum (AJ575812)* | 99.45 |
| 18 | W23-K81 | KR264984 | *Lactobacillus fermentum (MH041160)* | 99 | *Lactobacillus fermentum (*[*AJ575812*](https://www.ezbiocloud.net/16SrRNA?ac=AJ575812)*)* | 99.72 |
| 19 | W45-L80 | KR264983 | *Lactobacillus fermentum*  *(MH041171)* | 99 | *Lactobacillus fermentum (AJ575812)* | 99.68 |
| 20 | W20-K79 | KR264982 | *Streptococcus infantarius (MF355312)* | 99 | *Streptococcus infantarius (ABJK02000017)* | 99.33 |
| 21 | W5-K76 | KR264981 | *Lactobacillus mucosae (MF193456)* | 99 | *Lactobacillus mucosae (AF126738)* | 99.32 |
| 22 | W77-K530 | KT005522 | *Enterococcus faecalis (MH179309)* | 99 | *Enterococcus faecalis (ASDA0100000)* | 99.83 |
| 23 | W48-L527 | KT005521 | *Staphylococcus haemolyticus (*[*MH179468).*](https://www.ncbi.nlm.nih.gov/nucleotide/MH179468.1?report=genbank&log$=nucltop&blast_rank=2&RID=DVDD4UNP014) | 99 | *Staphylococcus haemolyticus* [*Yersinia pestis*](https://www.ezbiocloud.net/taxonomy?tn=Yersinia%20pestis)  [*X75274*](https://www.ezbiocloud.net/16SrRNA?ac=X75274) | 100 |
| 24 | W3-K533 | KT005520 | *Escherichia coli (MH197072)* | 100 | *Escherichia coli*  *(LN831047)* | 100 |
| 25 | W78-K528 | KT005519 | *Enterococcus faecalis (MH179309)* | 99 | *Enterococcus faecalis*  *(ASDA01000001)* | 99.62 |
| 26 | W66-K2 | KP747671 | *Janthinobacteriumlividum (KT767718)* | 99 | *Janthinobacteriumlividum (DQ355146)* | 99.87 |
| 27 | W5-L3 | KP747672 | *Staphylococcus epidermidis (MG966305)* | 100 | *Staphylococcus epidermidis*  *(L37605)* | 100 |
| 28 | W17-L17 | KP775934 | *Lactobacillus fermentum (LC037354)* | 99 | *Lactobacillus fermentum (AJ575812)* | 99.60 |
| 29 | W22-K16 | KP775933 | *Enterococcus faecium (MH127530)* | 100 | *Enterococcus faecium*  *(AJKH01000109)* | 100 |
| 30 | W71-K14 | KP775932 | *Staphylococcus hominis subsp. hominis (LT963442)* | 99 | *Staphylococcus hominis subsp. novobiosepticus*  *(AB233326)* | 100 |
| 31 | W20-K14 | KP775931 | *Enterococcus faecium (MH127530)* | 99 | *Enterococcus faecium*  *(AJKH01000109)* | 99.85 |
| 32 | W15-K12 | KP775929 | *Lactobacillus fermentum (LC037354)* | 99 | *Lactobacillus fermentum (AJ575812)* | 99.37 |
| 33 | W80-K11 | KP775928 | *Staphylococcus hominis (KY992547)* | 99 | *Staphylococcus hominis*  *(X66101)* | 99.23 |
| 34 | W19-K10 | KP775927 | *Lactobacillus gallinarum (*[*AB596996*](https://www.ncbi.nlm.nih.gov/nucleotide/AB596996.1?report=genbank&log$=nucltop&blast_rank=22&RID=DVGWHYXV014)*)* | 99 | *Lactobacillus gallinarum*  *(BALB01000057)* | 98.94 |
| 35 | W25-K9 | KP775926 | *Lactobacillus helveticus (*[*MG437355*](https://www.ncbi.nlm.nih.gov/nucleotide/MG437355.1?report=genbank&log$=nucltop&blast_rank=3&RID=DVH3CM8K015)*)* | 98 | *Lactobacillus helveticus (ACLM01000202)* | 97.17 |
| 36 | W57-K8 | KP775925 | *Lactobacillus gasseri (MH113149)* | 99 | *Lactobacillus gasseri (CP000413)* | 99.70 |
| 37 | W1-K7 | KP775924 | *Lactobacillus parafarraginis (*[*MG825721*](https://www.ncbi.nlm.nih.gov/nucleotide/MG825721.1?report=genbank&log$=nucltop&blast_rank=3&RID=DVHTPEST015)*)* | 97 | *Lactobacillus parafarraginis (AZFZ01000113)* | 97 |
| 38 | W42-K6 | KP775923 | *Lactobacillus fermentum (*[*LC037354*](https://www.ncbi.nlm.nih.gov/nucleotide/LC037354.1?report=genbank&log$=nucltop&blast_rank=2&RID=DVJ08FD0015)*)* | 99 | *Lactobacillus fermentum (*[*AJ575812*](https://www.ezbiocloud.net/16SrRNA?ac=AJ575812)*)* | 99.46 |
| 39 | W61-K5 | KP775922 | *Janthinobacteriumlividum (KT767718)* | 99 | *Janthinobacteriumlividum*  *(DQ355146)* | 99.87 |
| 40 | W39-KA115 | KT361204 | *Lactobacillus brevis (MH191230)* | 98 | *Lactobacillus brevis*  *(*[*KI271266*](https://www.ezbiocloud.net/16SrRNA?ac=KI271266)*)* | 98.44 |
| 41 | W32-K75 | KT361205 | *Weissellaconfusa (JQ754451)* | 97 | *Weissellaconfusa*  *(*[*AB023241*](https://www.ezbiocloud.net/16SrRNA?ac=AB023241)*)* | 99.38 |
| 42 | W11-K17 | KT361206 | *Staphylococcus epidermidis (MH014973)* | 99 | *Staphylococcus epidermidis (*[*L37605*](https://www.ezbiocloud.net/16SrRNA?ac=L37605)*)* | 100 |
| 43 | W16-K18 | KT361207 | *Staphylococcus epidermidis (*[*KY622647*](https://www.ncbi.nlm.nih.gov/nucleotide/KY622647.1?report=genbank&log$=nucltop&blast_rank=2&RID=DVJSN35G015)*)* | 99 | *Staphylococcus epidermidis (*[*L37605*](https://www.ezbiocloud.net/16SrRNA?ac=L37605)*)* | 99.77 |
| 44 | W20-K3 | KT361209 | *Lactobacillus helveticus (MG437355)* | 99 | *Lactobacillus helveticus (ACLM01000202)* | 97.87 |
| 45 | W37-K120 | KT361211 | *Lactobacillus helveticus (*[*MF942366*](https://www.ncbi.nlm.nih.gov/nucleotide/MF942366.1?report=genbank&log$=nucltop&blast_rank=4&RID=DVKVWG9Z014)*)* | 99 | *Lactobacillus helveticus (*[*ACLM01000202*](https://www.ezbiocloud.net/16SrRNA?ac=ACLM01000202)*)* | 98.82 |
| 46 | W19-K543 | KT589137 | *Enterococcus faecalis (*[*MH158265*](https://www.ncbi.nlm.nih.gov/nucleotide/MH158265.1?report=genbank&log$=nucltop&blast_rank=2&RID=DVM14G00014)*)* | 100 | *Enterococcus faecalis*  *(*[*ASDA01000001*](https://www.ezbiocloud.net/16SrRNA?ac=ASDA01000001)*)* | 100 |
| 47 | W17-K544 | KT589136 | *Enterococcus faecalis (KX073783)* | 100 | *Enterococcus faecalis*  *(ASDA01000001)* | 100 |
| 48 | W36-K548 | KT589135 | *Staphylococcus epidermidis (*[*MH111592*](https://www.ncbi.nlm.nih.gov/nucleotide/MH111592.1?report=genbank&log$=nucltop&blast_rank=2&RID=DVMCM38301R)*)* | 99 | *Staphylococcus epidermidis (*[*L37605*](https://www.ezbiocloud.net/16SrRNA?ac=L37605)*)* | 99.74 |
| 49 | W37-K123 | KT589133 | *Lactobacillus brevis (*[*LC199966*](https://www.ncbi.nlm.nih.gov/nucleotide/LC199966.1?report=genbank&log$=nucltop&blast_rank=2&RID=DVMMKB0Y014)*)* | 99 | *Lactobacillus brevis*  *(*[*KI271266*](https://www.ezbiocloud.net/16SrRNA?ac=KI271266)*)* | 99.74 |
| 50 | W33-K122 | KT589132 | *Lactobacillus brevis (GU132846)* | 99 | *Lactobacillus brevis*  *(*[*KI271266*](https://www.ezbiocloud.net/16SrRNA?ac=KI271266)*)* | 99.44 |
| 51 | W44-K547 | KT589131 | *Staphylococcus epidermidis (*[*KT589131*](https://www.ncbi.nlm.nih.gov/nucleotide/KT589131.1?report=genbank&log$=nucltop&blast_rank=15&RID=DVN32JXV014)*)* | 100 | *Staphylococcus epidermidis (*[*L37605*](https://www.ezbiocloud.net/16SrRNA?ac=L37605)*)* | 99.02 |
| 52 | W10-K121 | KT589130 | *Lactobacillus fermentum (*[*MF989239*](https://www.ncbi.nlm.nih.gov/nucleotide/MF989239.1?report=genbank&log$=nucltop&blast_rank=2&RID=DVN6JBSH01R)*)* | 99 | *Lactobacillus fermentum (*[*AJ575812*](https://www.ezbiocloud.net/16SrRNA?ac=AJ575812)*)* | 99.87 |
| 53 | W15-K545 | KT589128 | *Enterococcus faecalis (*[*KU726689*](https://www.ncbi.nlm.nih.gov/nucleotide/KU726689.1?report=genbank&log$=nucltop&blast_rank=3&RID=DVNJ0NNS014)*)* | 99 | *Enterococcus faecalis*  *(*[*ASDA01000001*](https://www.ezbiocloud.net/16SrRNA?ac=ASDA01000001)*)* | 99.74 |
| 54 | W43-K541 | KT589127 | *Staphylococcus epidermidis (*[*KY608100*](https://www.ncbi.nlm.nih.gov/nucleotide/KY608100.1?report=genbank&log$=nucltop&blast_rank=2&RID=DVNPEYDA014)*)* | 97 | *Staphylococcus epidermidis (*[*L37605*](https://www.ezbiocloud.net/16SrRNA?ac=L37605)*)* | 96.64 |
| 55 | W11-K540 | KT589126 | *Enterococcus faecalis (KM609191)* | 99 | *Enterococcus faecalis (*[*ASDA01000001*](https://www.ezbiocloud.net/16SrRNA?ac=ASDA01000001)*)* | 100 |
| 56 | W10-K539 | KT589125 | *Enterococcus faecalis (*[*KF826015*](https://www.ncbi.nlm.nih.gov/nucleotide/KF826015.1?report=genbank&log$=nucltop&blast_rank=4&RID=DVNYVBPR015)*)* | 100 | *Enterococcus faecalis*  *(*[*ASDA01000001*](https://www.ezbiocloud.net/16SrRNA?ac=ASDA01000001)*)* | 100 |
| 57 | W9-K538 | KT589124 | *Enterococcus faecalis (*[*MH158266*](https://www.ncbi.nlm.nih.gov/nucleotide/MH158266.1?report=genbank&log$=nucltop&blast_rank=1&RID=DVP4GKTJ015)*)* | 100 | *Enterococcus faecalis (*[*ASDA01000001*](https://www.ezbiocloud.net/16SrRNA?ac=ASDA01000001)*)* | 100 |
| 58 | W41-K537 | KT589123 | *Staphylococcus epidermidis (MH111592)* | 99 | *Staphylococcus epidermidis (*[*L37605*](https://www.ezbiocloud.net/16SrRNA?ac=L37605)*)* | 99.74 |
| 59 | W5-536 | KT589122 | *Enterococcus faecalis (*[*MH158266*](https://www.ncbi.nlm.nih.gov/nucleotide/MH158266.1?report=genbank&log$=nucltop&blast_rank=1&RID=DVPP51G201R)*)* | 100 | *Enterococcus faecalis (*[*ASDA01000001*](https://www.ezbiocloud.net/16SrRNA?ac=ASDA01000001)*)* | 100 |
| 60 | W44-K535 | KT589121 | *Escherichia coli (FJ789755)* | 99 | *Escherichia coli*  *(LN831047)* | 100 |
| 61 | W4-K534 | KT589120 | *Enterococcus faecalis (MH111613)* | 100 | *Enterococcus faecalis*  *(ASDA01000001)* | 100 |
| 62 | W1-K531 | KT589119 | *Enterococcus faecalis (MH179309)* | 99 | *Enterococcus faecalis (ASDA01000001)* | 99.74 |
| 63 | W57-K115 | KT589118 | *Enterococcus faecalis (MH158266)* | 99 | *Enterococcus faecalis (ASDA01000001)* | 99.87 |
| 64 | W55-K113 | KT589117 | *Lactobacillus brevis (*[*MF327676*](https://www.ncbi.nlm.nih.gov/nucleotide/MF327676.1?report=genbank&log$=nucltop&blast_rank=1&RID=DXH2M2VW015)*)* | 100 | *Lactobacillus brevis (*[*KI271266*](https://www.ezbiocloud.net/16SrRNA?ac=KI271266)*)* | 99.87 |
| 65 | W65-K112 | KT589116 | *Lactobacillus brevis (*[*MH191229*](https://www.ncbi.nlm.nih.gov/nucleotide/MH191229.1?report=genbank&log$=nucltop&blast_rank=1&RID=DXHMBE01015)*)* | 100 | *Lactobacillus brevis (*[*KI271266*](https://www.ezbiocloud.net/16SrRNA?ac=KI271266)*)* | 99.87 |
| 66 | W55-K111 | KT589115 | *Enterococcus faecalis (*[*MH158266.*](https://www.ncbi.nlm.nih.gov/nucleotide/MH158266.1?report=genbank&log$=nucltop&blast_rank=2&RID=DXHFZRN2014)*)* | 100 | *Enterococcus faecalis*  *(*[*ASDA01000001*](https://www.ezbiocloud.net/16SrRNA?ac=ASDA01000001)*)* | 99.87 |
| 67 | W54-K108 | KT589114 | *Enterococcus faecalis (*[*KF826015*](https://www.ncbi.nlm.nih.gov/nucleotide/KF826015.1?report=genbank&log$=nucltop&blast_rank=4&RID=DXHSH3A4014)*)* | 99 | *Enterococcus faecalis*  *(*[*ASDA01000001*](https://www.ezbiocloud.net/16SrRNA?ac=ASDA01000001)*)* | 99.76 |
| 68 | W54-KA105 | KT589113 | *Staphylococcus hominis (*[*MH141498*](https://www.ncbi.nlm.nih.gov/nucleotide/MH141498.1?report=genbank&log$=nucltop&blast_rank=4&RID=DXHY932P014)*)* | 99 | *Staphylococcus hominis sub novobiosepticus (*[*AB233326*](https://www.ezbiocloud.net/16SrRNA?ac=AB233326)*)* | 99.70 |
| 69 | W48-K102 | KT589112 | *Enterococcus faecalis (*[*KP297450*](https://www.ncbi.nlm.nih.gov/nucleotide/KP297450.1?report=genbank&log$=nucltop&blast_rank=2&RID=DXJ8PH4X015)*)* | 99 | *Enterococcus faecalis*  *(*[*ASDA01000001*](https://www.ezbiocloud.net/16SrRNA?ac=ASDA01000001)*)* | 99.75 |
| 70 | W47-K93 | KT589111 | *Enterococcus faecalis (*[*MH135230*](https://www.ncbi.nlm.nih.gov/nucleotide/MH135230.1?report=genbank&log$=nucltop&blast_rank=2&RID=DXJER0J8015)*)* | 99 | *Enterococcus faecalis (*[*ASDA01000001*](https://www.ezbiocloud.net/16SrRNA?ac=ASDA01000001)*)* | 99.87 |
| 71 | W43-K91 | KT589110 | *Enterococcus faecalis (*[*KT029322*](https://www.ncbi.nlm.nih.gov/nucleotide/KT029322.1?report=genbank&log$=nucltop&blast_rank=2&RID=DXJMPP0R015)*)* | 99 | *Enterococcus faecalis*  *(*[*ASDA01000001*](https://www.ezbiocloud.net/16SrRNA?ac=ASDA01000001)*)* | 99.64 |
| 72 | W42-K89 | KT589109 | *Enterococcus faecalis (*[*KX065363*](https://www.ncbi.nlm.nih.gov/nucleotide/KX065363.1?report=genbank&log$=nucltop&blast_rank=2&RID=DXKECRK9014)*)* | 99 | *Enterococcus faecalis*  *(*[*ASDA01000001*](https://www.ezbiocloud.net/16SrRNA?ac=ASDA01000001)*)* | 99.46 |
| 73 | W41-K84 | KT589108 | *Enterococcus faecalis (*[*MH158266*](https://www.ncbi.nlm.nih.gov/nucleotide/MH158266.1?report=genbank&log$=nucltop&blast_rank=5&RID=DXKTT3VK015)*)* | 98 | *Enterococcus faecalis*  *(*[*ASDA01000001*](https://www.ezbiocloud.net/16SrRNA?ac=ASDA01000001)*)* | 98.52 |
| 74 | W72-K83 | KT589107 | *Enterococcus faecalis (*[*HE646438*](https://www.ncbi.nlm.nih.gov/nucleotide/HE646438.1?report=genbank&log$=nucltop&blast_rank=5&RID=DXKYKY5M01R)*)* | 99 | *Enterococcus faecalis*  *(*[*ASDA01000001*](https://www.ezbiocloud.net/16SrRNA?ac=ASDA01000001)*)* | 99.86 |
| 75 | W3-K80 | KT589106 | *Lactobacillus fermentum (*[*KJ690754*](https://www.ncbi.nlm.nih.gov/nucleotide/KJ690754.1?report=genbank&log$=nucltop&blast_rank=3&RID=DXM6S6CN014)*)* | 99 | *Lactobacillus fermentum (*[*AJ575812*](https://www.ezbiocloud.net/16SrRNA?ac=AJ575812)*)* | 99.62 |
| 76 | W73-K77 | KT589105 | *Enterococcus faecalis (KF826015)* | 99 | *Enterococcus faecalis (*[*ASDA01000001*](https://www.ezbiocloud.net/16SrRNA?ac=ASDA01000001)*)* | 100 |
| 77 | W27-124 | KT597696 | *Lactobacillus mucosae (*[*MF975714*](https://www.ncbi.nlm.nih.gov/nucleotide/MF975714.1?report=genbank&log$=nucltop&blast_rank=3&RID=DXMTWS5G015)*)* | 96 | *Lactobacillus mucosae*  *(*[*AF126738*](https://www.ezbiocloud.net/16SrRNA?ac=AF126738)*)* | 95.87 |
| 78 | W17-K20 | KT597700 | *Janthinobacteriumlividum*  *(*[*KF318409*](https://www.ncbi.nlm.nih.gov/nucleotide/KF318409.1?report=genbank&log$=nucltop&blast_rank=11&RID=DXNK5EPZ014)*)* | 96 | *Janthinobacterium lividum (*[*HG322949*](https://www.ezbiocloud.net/16SrRNA?ac=HG322949)*)* | 96.89 |
| 79 | W32-A4 | KT835007 | *Lactobacillus mucosae (*[*MF975714.*](https://www.ncbi.nlm.nih.gov/nucleotide/MF975714.1?report=genbank&log$=nucltop&blast_rank=6&RID=DXNSWU3H015)*)* | 99 | *Lactobacillus mucosae (*[*AF126738*](https://www.ezbiocloud.net/16SrRNA?ac=AF126738)*)* | 99.86 |
| 80 | W9-A5 | KT835008 | *Lactobacillus mucosae (*[*MF989244*](https://www.ncbi.nlm.nih.gov/nucleotide/MF989244.1?report=genbank&log$=nucltop&blast_rank=3&RID=DXNY3ZAZ014)*)* | 99 | *Lactobacillus mucosae (*[*AF126738*](https://www.ezbiocloud.net/16SrRNA?ac=AF126738)*)* | 99.86 |
| 81 | W1-A6 | KT835009 | *Lactobacillus mucosae (*[*MF975714*](https://www.ncbi.nlm.nih.gov/nucleotide/MF975714.1?report=genbank&log$=nucltop&blast_rank=6&RID=DXP3MHDB015)*)* | 99 | *Lactobacillus mucosae (*[*AF126738*](https://www.ezbiocloud.net/16SrRNA?ac=AF126738)*)* | 99.73 |
| 82 | W33-A7 | KT835010 | *Lactobacillus mucosae (*[*MG966335*](https://www.ncbi.nlm.nih.gov/nucleotide/MG966335.1?report=genbank&log$=nucltop&blast_rank=4&RID=DXPASKUM015)*)* | 99 | *Lactobacillus mucosae (*[*AF126738*](https://www.ezbiocloud.net/16SrRNA?ac=AF126738)*)* | 100 |
| 83 | W67-A8 | KT835011 | *Lactobacillus mucosae (MG966335)* | 99 | *Lactobacillus mucosae (*[*AF126738*](https://www.ezbiocloud.net/16SrRNA?ac=AF126738)*)* | 99.87 |
| 84 | W54-A9 | KT835012 | *Lactobacillus mucosae (*[*MG966335*](https://www.ncbi.nlm.nih.gov/nucleotide/MG966335.1?report=genbank&log$=nucltop&blast_rank=1&RID=DXRN8668014)*)* | 100 | *Lactobacillus mucosae*  *(*[*AF126738*](https://www.ezbiocloud.net/16SrRNA?ac=AF126738)*)* | 100 |
| 85 | W58-A10 | KT835013 | *Lactobacillus mucosae (*[*MG966335*](https://www.ncbi.nlm.nih.gov/nucleotide/MG966335.1?report=genbank&log$=nucltop&blast_rank=6&RID=DXRU0GAP01R)*)* | 99 | *Lactobacillus mucosae*  *(*[*AF126738*](https://www.ezbiocloud.net/16SrRNA?ac=AF126738)*)* | 99.87 |
| 86 | W60-AN12 | KT835014 | *Streptococcus anginosus (JN787180)* | 99 | *Streptococcus constellatus subsp. viborgensis (JN787160)* | 98.78 |
| 87 | W56-A13 | KT835015 | *Lactobacillus mucosae (*[*MG966335*](https://www.ncbi.nlm.nih.gov/nucleotide/MG966335.1?report=genbank&log$=nucltop&blast_rank=6&RID=DXSKV90M015)*)* | 99 | *Lactobacillus mucosae*  *(*[*AF126738*](https://www.ezbiocloud.net/16SrRNA?ac=AF126738)*)* | 99.87 |
| 88 | W40-A15 | KT835016 | *Streptococcus gallolyticus*  *(*[*LC269371*](https://www.ncbi.nlm.nih.gov/nucleotide/LC269371.1?report=genbank&log$=nucltop&blast_rank=15&RID=DXSV2R74015)*)* | 99 | *Streptococcus gallolyticus (*[*FOLZ01000015*](https://www.ezbiocloud.net/16SrRNA?ac=FOLZ01000015)*)* | 99.87 |
| 89 | W70-A16 | KT835017 | *Streptococcus gallolyticus (LC269371)* | 99 | *Streptococcus gallolyticus (FOLZ01000015)* | 99.87 |
| 90 | W57-A20 | KT835018 | *Lactobacillus mucosae (MF975714)* | 99 | *Lactobacillus mucosae*  *(AF126738)* | 99.87 |
| 91 | W63-AN1 | KT906568 | *Streptococcus anginosus (*[*AF145246*](https://www.ncbi.nlm.nih.gov/nucleotide/AF145246.1?report=genbank&log$=nucltop&blast_rank=5&RID=DXTU2TBH015)*)* | 99 | [*Streptococcus anginosus subsp. whileyi*](https://www.ezbiocloud.net/taxonomy?tn=Streptococcus%20anginosus%20subsp.%20whileyi)  *(JN787193)* | 97.91 |
| 92 | W82-AN2 | KT906569 | *Staphylococcus capitis (MF033474)* | 99 | *Staphylococcus capitis subsp. urelyticus*  *(AB233325)* | 99.47 |
| 93 | W58-AN3 | KT906570 | *Streptococcus anginosus (KX661075)* | 99 | *Streptococcus anginosus (JN787193)* | 99.52 |
| 94 | W7-AN4 | KT906571 | *Propionibacteriumavidum*  *(KX959616)*  *Cutibacteriumavidum*  *(MH201146)* | 99 | [*Cutibacterium avidum*](https://www.ezbiocloud.net/taxonomy?tn=Cutibacterium%20avidum) *(AGBA01000019)* | 99.87 |
| 95 | W25-AN6 | KT906572 | *Enterococcus faecalis (*[*MH158266*](https://www.ncbi.nlm.nih.gov/nucleotide/MH158266.1?report=genbank&log$=nucltop&blast_rank=2&RID=DXUVV1UY014)*)* | 99 | *Enterococcus faecalis (ASDA01000001)* | 99.28 |
| 96 | W27-AN7 | KT906573 | *Enterococcus faecalis (MH111642)* | 100 | *Enterococcus faecalis*  *(ASDA01000001)* | 100 |
| 97 | W83-AN8 | KT906574 | *Streptococcus agalactiae (*[*CP025028*](https://www.ncbi.nlm.nih.gov/nucleotide/CP025028.1?report=genbank&log$=nucltop&blast_rank=1&RID=DXV5S3P7015)*)* | 100 | *Streptococcus agalactiae*  *(*[*AEQQ01000116*](https://www.ezbiocloud.net/16SrRNA?ac=AEQQ01000116)*)* | 99.88 |
| 98 | W79-AN9 | KT906575 | *Streptococcus agalactiae (*[*CP020449*](https://www.ncbi.nlm.nih.gov/nucleotide/CP020449.2?report=genbank&log$=nucltop&blast_rank=1&RID=DXVAB35J014)*)* | 100 | *Streptococcus agalactiae (AEQQ01000116)* | 100 |
| 99 | W28-AN10 | KT906576 | *Lactobacillus jensenii (KF740715)* | 100 | *Lactobacillus jensenii*  *(AYYU01000057)* | 100 |
| 100 | W7-AN11 | KT906577 | *Bifidobacteriumbreve (*[*CP023198*](https://www.ncbi.nlm.nih.gov/nucleotide/CP023198.1?report=genbank&log$=nucltop&blast_rank=2&RID=DXVVHXEJ015)*)* |  | *Bifidobacteriumbreve (*[*ACCG01000002*](https://www.ezbiocloud.net/16SrRNA?ac=ACCG01000002)*)* | 99.68 |
| 101 | W16-AN12 | KT906578 | *Lactobacillus jensenii (*[*CP018809*](https://www.ncbi.nlm.nih.gov/nucleotide/CP018809.1?report=genbank&log$=nucltop&blast_rank=2&RID=DXW5GXHJ014)*)* | 99 | *Lactobacillus jensenii (*[*AYYU01000057*](https://www.ezbiocloud.net/16SrRNA?ac=AYYU01000057)*)* | 100 |
| 102 | W34-AN13 | KT906579 | *Bifidobacteriumbreve (CP023198)* | 100 | *Bifidobacteriumbreve (*[*ACCG01000002*](https://www.ezbiocloud.net/16SrRNA?ac=ACCG01000002)*)* | 99.88 |
| 103 | W61-AN14 | KT906580 | *Bifidobacteriumbreve (*[*CP023198*](https://www.ncbi.nlm.nih.gov/nucleotide/CP023198.1?report=genbank&log$=nucltop&blast_rank=2&RID=DXWRYJEU014)*)* | 99 | *Bifidobacteriumbreve (*[*ACCG01000002*](https://www.ezbiocloud.net/16SrRNA?ac=ACCG01000002)*)* | 99.13 |
| 104 | W26-AN18 | KT906581 | *Propionibacteriumavidum (*[*KU975425*](https://www.ncbi.nlm.nih.gov/nucleotide/KU975425.1?report=genbank&log$=nucltop&blast_rank=3&RID=DXWWN87E015)*)* | 100 | [*Cutibacterium avidum*](https://www.ezbiocloud.net/taxonomy?tn=Cutibacterium%20avidum)  *(AGBA01000019)* | 100 |
| 105 | W29- AN19 | KT906582 | *Propionibacteriumavidum*  *(KU975425)*  *(KX646258)* | 99 | [*Cutibacterium avidum*](https://www.ezbiocloud.net/taxonomy?tn=Cutibacterium%20avidum)  *(AGBA01000019)* | 99.87 |
| 106 | W35-AN20 | KT906583 | *Propionibacteriumavidum (*[*KU975425*](https://www.ncbi.nlm.nih.gov/nucleotide/KU975425.1?report=genbank&log$=nucltop&blast_rank=4&RID=DXXCTAJH015)*)* | 100 | [*Cutibacterium avidum*](https://www.ezbiocloud.net/taxonomy?tn=Cutibacterium%20avidum) *(*[*AGBA01000019*](https://www.ezbiocloud.net/16SrRNA?ac=AGBA01000019)*)* | 100 |
| 107 | W45-AN24 | KT906584 | *Propionibacteriumavidum (*[*KU975425*](https://www.ncbi.nlm.nih.gov/nucleotide/KU975425.1?report=genbank&log$=nucltop&blast_rank=4&RID=DY3HB997014)*)* | 100 | [*Cutibacterium avidum*](https://www.ezbiocloud.net/taxonomy?tn=Cutibacterium%20avidum) *(*[*AGBA01000019*](https://www.ezbiocloud.net/16SrRNA?ac=AGBA01000019)*)* | 100 |
| 108 | W29- AN25 | KT906585 | *Kocuriakristinae (MG948160)* | 100 | *Kocuriakristinae*  *(BCSM0100002)* | 100 |
| 109 | W74-AN26 | KT906586 | *Staphylococcus kloosii (*[*HM355640*](https://www.ncbi.nlm.nih.gov/nucleotide/HM355640.1?report=genbank&log$=nucltop&blast_rank=18&RID=DY42D3CJ014)*)* | 99 | *Staphylococcus kloosii*  *(*[*AB009940*](https://www.ezbiocloud.net/16SrRNA?ac=AB009940)*)* | 99.87 |
| 110 | W83-AN30 | KT906587 | *Enterococcus faecalis (*[*MF370045*](https://www.ncbi.nlm.nih.gov/nucleotide/MF370045.1?report=genbank&log$=nucltop&blast_rank=2&RID=DY47XAX9014)*)* | 99 | *Enterococcus faecalis (*[*ASDA01000001*](https://www.ezbiocloud.net/16SrRNA?ac=ASDA01000001)*)* | 99.76 |
| 111 | W38-AN31 | KT906588 | *Lactobacillus jensenii (*[*CP018809*](https://www.ncbi.nlm.nih.gov/nucleotide/CP018809.1?report=genbank&log$=nucltop&blast_rank=1&RID=DY4D2XB5015)*)* | 100 | *Lactobacillus jensenii*  *(*  [*AYYU01000057*](https://www.ezbiocloud.net/16SrRNA?ac=AYYU01000057)  *)* | 100 |
| 112 | W67-AN22 | KT906589 | *Streptococcus agalactiae strain AN22*  *(*  [*CP018809*](https://www.ncbi.nlm.nih.gov/nucleotide/CP018809.1?report=genbank&log$=nucltop&blast_rank=1&RID=DY4SM5KR015)*)*  *L. jensenii* |  | *Streptococcus agalactiae (*  [*AYYU01000057*](https://www.ezbiocloud.net/16SrRNA?ac=AYYU01000057)*)*  *L. jensenii* |  |
| 113 | W75-AN35 | KT991842 | *Lactobacillus gasseri (*[*MH111639*](https://www.ncbi.nlm.nih.gov/nucleotide/MH111639.1?report=genbank&log$=nucltop&blast_rank=1&RID=DY557RB0014)*)* | 100 | *Lactobacillus gasseri (*[*CP000413*](https://www.ezbiocloud.net/16SrRNA?ac=CP000413)*)* | 99.87 |
| 114 | W77-AN36 | KT991843 | *Lactobacillus gasseri (*[*MH111639*](https://www.ncbi.nlm.nih.gov/nucleotide/MH111639.1?report=genbank&log$=nucltop&blast_rank=3&RID=DY7BARJ7014)*)* | 99 | *Lactobacillus gasseri (*[*CP000413*](https://www.ezbiocloud.net/16SrRNA?ac=CP000413)*)* | 99.20 |
| 115 | W11-AN54 | KT991844 | *Lactobacillus mucosae (*[*MG966335*](https://www.ncbi.nlm.nih.gov/nucleotide/MG966335.1?report=genbank&log$=nucltop&blast_rank=2&RID=DY7N01WF015)*)* | 100 | *Lactobacillus mucosae (*[*AF126738*](https://www.ezbiocloud.net/16SrRNA?ac=AF126738)*)* | 100 |
| 116 | W31-AN5 | KU184460 | *Enterococcus faecalis (*[*JN644614*](https://www.ncbi.nlm.nih.gov/nucleotide/JN644614.1?report=genbank&log$=nucltop&blast_rank=2&RID=DY7U57MK014)*)* | 99 | *Enterococcus faecalis*  *(*[*ASDA01000001*](https://www.ezbiocloud.net/16SrRNA?ac=ASDA01000001)*)* | 99.74 |
| 117 | W33-AN42 | KU184461 | *Enterococcus faecalis (*[*MH179309*](https://www.ncbi.nlm.nih.gov/nucleotide/MH179309.1?report=genbank&log$=nucltop&blast_rank=1&RID=DY7Z4XH6015)*)* | 100 | *Enterococcus faecalis*  *(*[*ASDA01000001*](https://www.ezbiocloud.net/16SrRNA?ac=ASDA01000001)*)* | 100 |
| 118 | W36-AN44 | KU184462 | *Enterococcus faecalis (*[*KF250867.*](https://www.ncbi.nlm.nih.gov/nucleotide/KF250867.1?report=genbank&log$=nucltop&blast_rank=2&RID=DY834EZA014)*)* | 99 | *Enterococcus faecalis*  *(*[*ASDA01000001*](https://www.ezbiocloud.net/16SrRNA?ac=ASDA01000001)*)* | 99.47 |
| 119 | W80-AN46 | KU184463 | *Lactobacillus gasseri (*[*MH111639*](https://www.ncbi.nlm.nih.gov/nucleotide/MH111639.1?report=genbank&log$=nucltop&blast_rank=7&RID=DY89HRFB014)*)* | 99 | *Lactobacillus gasseri*  *(*[*CP000413*](https://www.ezbiocloud.net/16SrRNA?ac=CP000413)*)* | 99.65 |
| 120 | W37-AN47 | KU184464 | *Enterococcus faecalis (*[*MH158266*](https://www.ncbi.nlm.nih.gov/nucleotide/MH158266.1?report=genbank&log$=nucltop&blast_rank=1&RID=DY8DYZFU015)*)* | 100 | *Enterococcus faecalis*  *(*[*ASDA01000001*](https://www.ezbiocloud.net/16SrRNA?ac=ASDA01000001)*)* | 100 |
| 121 | W38-AN49 | KU184465 | *Enterococcus faecalis (*[*MH158266*](https://www.ncbi.nlm.nih.gov/nucleotide/MH158266.1?report=genbank&log$=nucltop&blast_rank=1&RID=DY8HJTST014)*)* | 100 | *Enterococcus faecalis*  *(*  [*ASDA01000001*](https://www.ezbiocloud.net/16SrRNA?ac=ASDA01000001)  *)* | 100 |
| 122 | W81-AN50 | KU184466 | *Lactobacillus gasseri (*[*MH111639*](https://www.ncbi.nlm.nih.gov/nucleotide/MH111639.1?report=genbank&log$=nucltop&blast_rank=7&RID=DY8NC1PK014)*)* | 99 | *Lactobacillus gasseri*  *(*[*CP000413*](https://www.ezbiocloud.net/16SrRNA?ac=CP000413)*)* | 99.65 |
| 123 | W73-AN57 | KU184467 | *Lactobacillus mucosae (*[*MG966335*](https://www.ncbi.nlm.nih.gov/nucleotide/MG966335.1?report=genbank&log$=nucltop&blast_rank=2&RID=DY8T4ARA015)*)* | 99 | *Lactobacillus mucosae*  *(*  [*AF126738*](https://www.ezbiocloud.net/16SrRNA?ac=AF126738)*)* | 100 |
| 124 | W51-AN58 | KU184468 | *Lactobacillus mucosae (*  [*MG966335*](https://www.ncbi.nlm.nih.gov/nucleotide/MG966335.1?report=genbank&log$=nucltop&blast_rank=1&RID=DY8WWFPX015)*)* | 100 | *Lactobacillus mucosae*  *(*[*AF126738*](https://www.ezbiocloud.net/16SrRNA?ac=AF126738)*)* | 100 |
| 125 | W13-AN62 | KU184469 | *Enterococcus faecalis (*[*MH111642*](https://www.ncbi.nlm.nih.gov/nucleotide/MH111642.1?report=genbank&log$=nucltop&blast_rank=1&RID=DY947W44014)*)* | 100 | *Enterococcus faecalis*  *(*  [*ASDA01000001*](https://www.ezbiocloud.net/16SrRNA?ac=ASDA01000001)*)* | 100 |
| 126 | W3-AN67 | KU184470 | *Enterococcus faecalis (*[*MH111642*](https://www.ncbi.nlm.nih.gov/nucleotide/MH111642.1?report=genbank&log$=nucltop&blast_rank=1&RID=DY986BT4015)*)* | 100 | *Enterococcus faecalis*  *(*  [*ASDA01000001*](https://www.ezbiocloud.net/16SrRNA?ac=ASDA01000001)*)* | 100 |
| 127 | W50-AN68 | KU184471 | *Enterococcus faecalis (*[*MH158266*](https://www.ncbi.nlm.nih.gov/nucleotide/MH158266.1?report=genbank&log$=nucltop&blast_rank=4&RID=DY9F1N45015)*)* | 99 | *Enterococcus faecalis*  *(*[*ASDA01000001*](https://www.ezbiocloud.net/16SrRNA?ac=ASDA01000001)*)* | 98.86 |
| 128 | W60-AN69 | KU184472 | *Enterococcus faecalis (*[*MH158266*](https://www.ncbi.nlm.nih.gov/nucleotide/MH158266.1?report=genbank&log$=nucltop&blast_rank=3&RID=E05FWA36014)*)* | 99 | *Enterococcus faecalis*  *(*[*ASDA01000001*](https://www.ezbiocloud.net/16SrRNA?ac=ASDA01000001)*)* | 98.79 |
| 129 | W13-AN70 | KU184473 | *Lactobacillus fermentum (*  [*KF148876*](https://www.ncbi.nlm.nih.gov/nucleotide/KF148876.1?report=genbank&log$=nucltop&blast_rank=2&RID=E05NV5NY014)  *)* | 100 | *Lactobacillus fermentum (*[*AJ575812*](https://www.ezbiocloud.net/16SrRNA?ac=AJ575812)*)* | 99.98 |
| 130 | W65-AN78 | KU184474 | *Enterococcus faecalis (*  [*MH158266*](https://www.ncbi.nlm.nih.gov/nucleotide/MH158266.1?report=genbank&log$=nucltop&blast_rank=1&RID=E05W7M8E014)  *)* | 100 | *Enterococcus faecalis*  *(*[*ASDA01000001*](https://www.ezbiocloud.net/16SrRNA?ac=ASDA01000001)*)* | 100 |
| 131 | W41-AN79 | KU184475 | *Lactobacillus ingluviei (*[*KR492882*](https://www.ncbi.nlm.nih.gov/nucleotide/KR492882.1?report=genbank&log$=nucltop&blast_rank=2&RID=E06331HS01R)*)* | 99 | *Lactobacillus ingluviei*  *(*[*AZFK01000041*](https://www.ezbiocloud.net/16SrRNA?ac=AZFK01000041)*)* | 99.76 |
| 132 | W56-K69 | KU184476 | *Enterococcus hirae (*[*MF327681*](https://www.ncbi.nlm.nih.gov/nucleotide/MF327681.1?report=genbank&log$=nucltop&blast_rank=1&RID=E066BZUZ01R)*)* | 100 | *Enterococcus hirae*  *(*[*CP003504*](https://www.ezbiocloud.net/16SrRNA?ac=CP003504)*)* | 100 |
| 133 | W51-K70 | KU184477 | *Enterococcus hirae (*[*MF327681*](https://www.ncbi.nlm.nih.gov/nucleotide/MF327681.1?report=genbank&log$=nucltop&blast_rank=1&RID=E06BC42U014)*)* | 100 | *Enterococcus hirae (*[*CP003504*](https://www.ezbiocloud.net/16SrRNA?ac=CP003504)*)* | 100 |
| 134 | W45-K86 | KU184478 | *Enterococcus hirae (*[*MF327669*](https://www.ncbi.nlm.nih.gov/nucleotide/MF327669.1?report=genbank&log$=nucltop&blast_rank=1&RID=E06FARZF015)*)* | 100 | *Enterococcus hirae (*[*CP003504*](https://www.ezbiocloud.net/16SrRNA?ac=CP003504)*)* | 100 |
| 135 | W28- K92 | KU184479 | *Enterococcus faecalis (*  [*MH158266*](https://www.ncbi.nlm.nih.gov/nucleotide/MH158266.1?report=genbank&log$=nucltop&blast_rank=1&RID=E06SNN4K015)*)* | 100 | *Enterococcus faecalis \*  *(*[*ASDA01000001*](https://www.ezbiocloud.net/16SrRNA?ac=ASDA01000001)*)* | 100 |
| 136 | W47-L102 | KU184480 | *Enterococcus hirae (*[*MF327681*](https://www.ncbi.nlm.nih.gov/nucleotide/MF327681.1?report=genbank&log$=nucltop&blast_rank=1&RID=E072AKPP014)*)* | 100 | *Enterococcus hirae (*  [*CP003504*](https://www.ezbiocloud.net/16SrRNA?ac=CP003504)*)* | 100 |
| 137 | W74-L125 | KU184483 | *Lactobacillus mucosae (*[*MF975714*](https://www.ncbi.nlm.nih.gov/nucleotide/MF975714.1?report=genbank&log$=nucltop&blast_rank=6&RID=E07C5399015)*)* | 99 | *Lactobacillus mucosae (*[*AF126738*](https://www.ezbiocloud.net/16SrRNA?ac=AF126738)*)* | 99.86 |
| 138 | W78-K126 | KU184484 | *Lactobacillus mucosae (*[*MF975714*](https://www.ncbi.nlm.nih.gov/nucleotide/MF975714.1?report=genbank&log$=nucltop&blast_rank=8&RID=E07HJFCE014)*)* | 99 | *Lactobacillus mucosae*  *(*[*AF126738*](https://www.ezbiocloud.net/16SrRNA?ac=AF126738)*)* | 99.86 |
| 139 | W82-K127 | KU184485 | *Lactobacillus mucosae (*[*MG966335.*](https://www.ncbi.nlm.nih.gov/nucleotide/MG966335.1?report=genbank&log$=nucltop&blast_rank=2&RID=E07RBKT1014)*)* | 99 | *Lactobacillus mucosae*  *(*[*AF126738*](https://www.ezbiocloud.net/16SrRNA?ac=AF126738)*)* | 99.86 |
| 140 | W83-K128 | KU184486 | *Lactobacillus mucosae (*[*MF975714*](https://www.ncbi.nlm.nih.gov/nucleotide/MF975714.1?report=genbank&log$=nucltop&blast_rank=3&RID=E07VBECP01R)*)* | 99 | *Lactobacillus mucosae*  *(*[*AF126738*](https://www.ezbiocloud.net/16SrRNA?ac=AF126738)*)* | 99.86 |
| 141 | W4-L129 | KU184487 | *Lactobacillus mucosae (*[*MF975714*](https://www.ncbi.nlm.nih.gov/nucleotide/MF975714.1?report=genbank&log$=nucltop&blast_rank=11&RID=E080SMUV014)*)* | 99 | *Lactobacillus mucosae (*[*AF126738*](https://www.ezbiocloud.net/16SrRNA?ac=AF126738)*)* | 99.86 |
| 142 | W56-K130 | KU184488 | *Lactobacillus mucosae (*[*MF975714.*](https://www.ncbi.nlm.nih.gov/nucleotide/MF975714.1?report=genbank&log$=nucltop&blast_rank=7&RID=E086ZD2R015)*)* | 99 | *Lactobacillus mucosae*  *(*[*AF126738*](https://www.ezbiocloud.net/16SrRNA?ac=AF126738)*)* | 99.86 |
| 143 | W47-K131 | KU184489 | *Lactobacillus mucosae (*[*MG966335*](https://www.ncbi.nlm.nih.gov/nucleotide/MG966335.1?report=genbank&log$=nucltop&blast_rank=6&RID=E08GTGGP01R)*)* | 99 | *Lactobacillus mucosae*  *(*[*AF126738*](https://www.ezbiocloud.net/16SrRNA?ac=AF126738)*)* | 99.87 |
| 144 | W43-K132 | KU184490 | *Lactobacillus mucosae (*[*MG966335*](https://www.ncbi.nlm.nih.gov/nucleotide/MG966335.1?report=genbank&log$=nucltop&blast_rank=6&RID=E08TGHCK015)*)* | 99 | *Lactobacillus mucosae*  *(*[*AF126738*](https://www.ezbiocloud.net/16SrRNA?ac=AF126738)*)* | 99.87 |
| 145 | W48-K133 | KU184491 | *Lactobacillus mucosae (*[*MG966335*](https://www.ncbi.nlm.nih.gov/nucleotide/MG966335.1?report=genbank&log$=nucltop&blast_rank=1&RID=E08ZT2B7015)*)* | 100 | *Lactobacillus mucosae*  *(*[*AF126738*](https://www.ezbiocloud.net/16SrRNA?ac=AF126738)*)* | 100 |
| 146 | W38-AN23 | KU184493 | *Propionibacterium avidum*  *(*[*CP016954*](https://www.ncbi.nlm.nih.gov/nucleotide/CP016954.1?report=genbank&log$=nucltop&blast_rank=3&RID=E092ZRZW014)*)* | 100 | *Propionibacterium avidum*  *(*[*AGBA01000019*](https://www.ezbiocloud.net/16SrRNA?ac=AGBA01000019)*)* | 100 |
| 147 | W13-A27 | KU184494 | *Bacillus subtilis (*[*MH045986*](https://www.ncbi.nlm.nih.gov/nucleotide/MH045986.1?report=genbank&log$=nucltop&blast_rank=2&RID=E09BXW82014)*)* | 100 | *Bacillus subtilis*  *(*[*ABQL01000001*](https://www.ezbiocloud.net/16SrRNA?ac=ABQL01000001)*)* | 100 |
| 148 | W63-AN59 | KU184495 | *Lactobacillus mucosae (*[*AF126738*](https://www.ezbiocloud.net/16SrRNA?ac=AF126738)*)* | 100 | *Lactobacillus mucosae*  *(*[*MG966335*](https://www.ncbi.nlm.nih.gov/nucleotide/MG966335.1?report=genbank&log$=nucltop&blast_rank=1&RID=E09JCCM2015)*)* | 100 |
| 149 | W69-AN64 | KU184496 | *Enterococcus faecalis (*[*MH111642*](https://www.ncbi.nlm.nih.gov/nucleotide/MH111642.1?report=genbank&log$=nucltop&blast_rank=1&RID=E09RF2YJ014)*)* | 100 | *Enterococcus faecalis*  *(*[*ASDA01000001*](https://www.ezbiocloud.net/16SrRNA?ac=ASDA01000001)*)* | 100 |
| 150 | W52-AN66 | KU184497 | *Lactobacillus mucosae (*[*EU547775*](https://www.ncbi.nlm.nih.gov/nucleotide/EU547775.1?report=genbank&log$=nucltop&blast_rank=2&RID=E0A0040M014)*)* | 99 | *Lactobacillus mucosae (*[*AF126738*](https://www.ezbiocloud.net/16SrRNA?ac=AF126738)*)* | 99.60 |
| 151 | W70-AN72 | KU184498 | *Enterococcus faecalis (*[*EU547775*](https://www.ncbi.nlm.nih.gov/nucleotide/EU547775.1?report=genbank&log$=nucltop&blast_rank=2&RID=E0A4EN3E014)*)* | 99 | *Enterococcus faecalis*  *(ASDA01000001)* | 99.86 |
| 152 | W62-AN74 | KU184499 | *Lactobacillus fermentum*  *(*[*MG551128*](https://www.ncbi.nlm.nih.gov/nucleotide/MG551128.1?report=genbank&log$=nucltop&blast_rank=1&RID=E0AH8Y68014)*)* | 100 | *Lactobacillus fermentum (*[*AJ575812*](https://www.ezbiocloud.net/16SrRNA?ac=AJ575812)*)* | 99.87 |
| 153 | W39-K79 | KU184500 | *Lactobacillus ingluviei (*[*KR492882*](https://www.ncbi.nlm.nih.gov/nucleotide/KR492882.1?report=genbank&log$=nucltop&blast_rank=3&RID=E0APCTS0014)*)* | 99 | *Lactobacillus ingluviei*  *(*[*AZFK01000041*](https://www.ezbiocloud.net/16SrRNA?ac=AZFK01000041)*)* | 99.88 |
| 154 | W36-K105A | KR264995 | *Staphylococcus hominis subsp. hominis (*[*MH174446*](https://www.ncbi.nlm.nih.gov/nucleotide/MH174446.1?report=genbank&log$=nucltop&blast_rank=2&RID=E0AUT3XC015)*)* | 99 | *Staphylococcus hominis subsp. hominis (*[*X66101*](https://www.ezbiocloud.net/16SrRNA?ac=X66101)*)* | 99.76 |
