# Supplementary figures and images for: Contrasting diversity of vaginal lactobacilli among the females of Northeast India
Source: BMC Microbiol. 2019 Aug 27;19:198. doi: 10.1186/s12866-019-1568-6 (PMC6712660; doi:10.1186/s12866-019-1568-6)

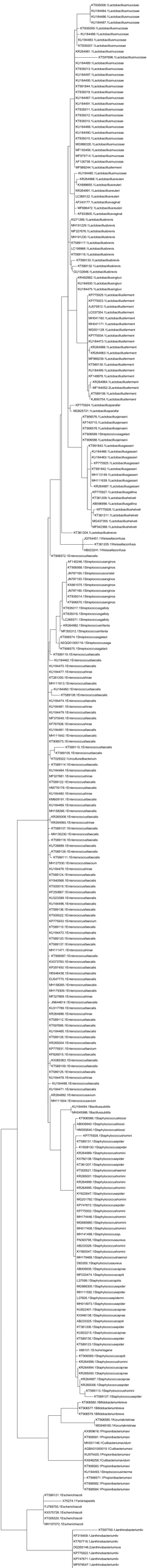

0.05

Supplement: Supplementary file 6 — Figure S1. Maximum Likelihood based clustering of developed and database sequences. The sequences of same species clustered together and distinct with respect to sequences of other species. (PDF 50 kb) [file 12866_2019_1568_MOESM6_ESM.pdf]

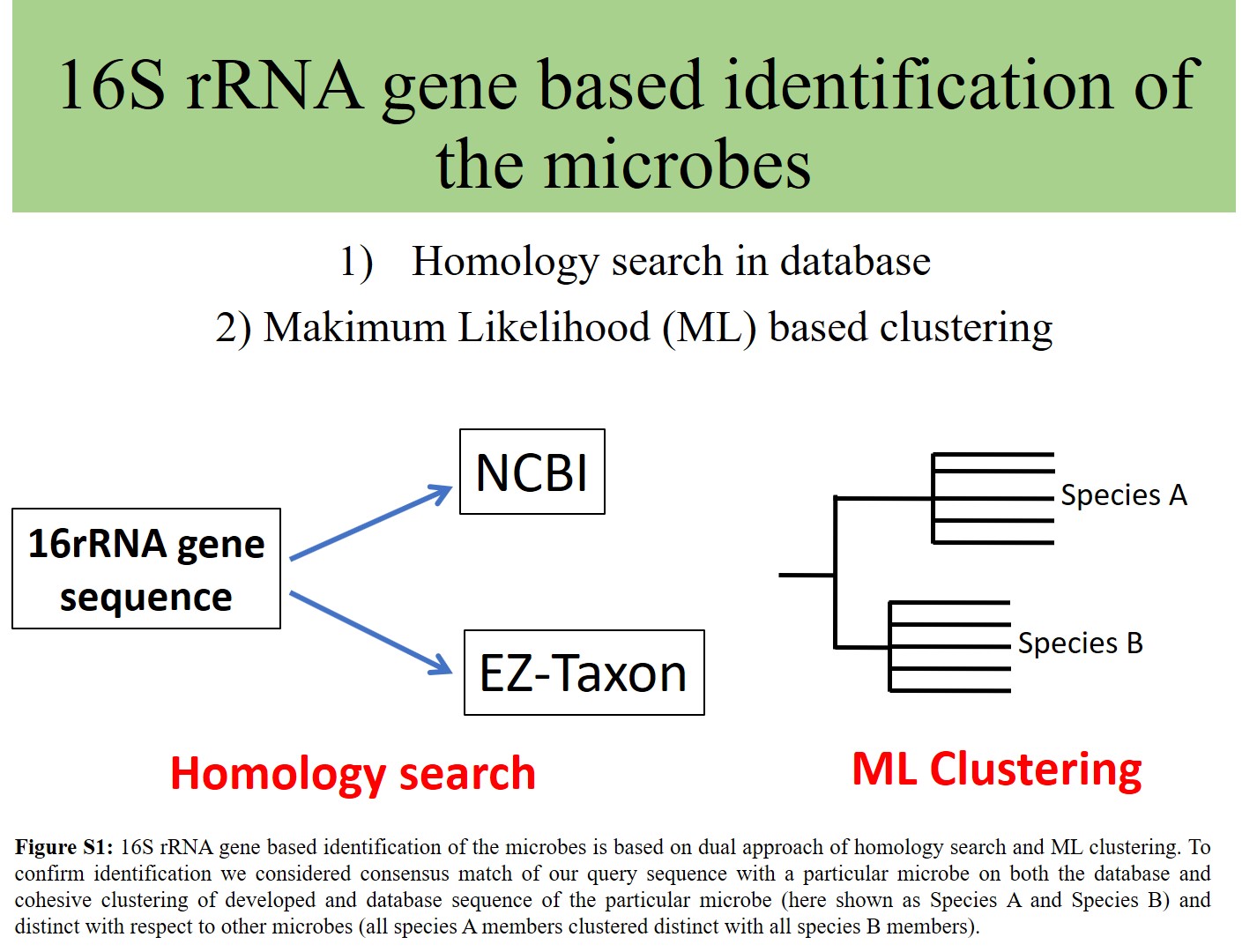

Supplement: Supplementary file 7 — Figure S2. 16S rRNA gene based identification of the microbes is based on dual approach of homology search and ML clustering. To confirm identification we considered consensus match of our query sequence with a particular microbe on both the database and cohesive clustering of developed and database sequence of the particular microbe (here shown as Species A and Species B) and distinct with respect to other microbes (all species A members clustered distinct with all species B members). (JPG 250 kb) [file 12866_2019_1568_MOESM7_ESM.jpg]
